# REEP5 mediates the function of CLEC5A to alleviate myocardial infarction by inhibiting endoplasmic reticulum stress-induced apoptosis

**DOI:** 10.1186/s12872-024-04018-3

**Published:** 2024-07-23

**Authors:** Xin Wang, Limin Sun

**Affiliations:** 1 Department of Cardiology, The First Affiliated Hospital of Jinzhou Medical University, Jinzhou, China; 2 Department of General Practice, The First Affiliated Hospital of Jinzhou Medical University, Jinzhou, China

**Keywords:** Myocardial infarction, REEP5, CLEC5A, Endoplasmic reticulum stress, Apoptosis

## Abstract

MI (myocardial infarction) often triggers severe heart failure and is one of the leading causes of death worldwide. Receptor expression-enhancing protein 5 (REEP5), a member of REEPs, acts as regulators of endoplasmic reticulum (ER) affecting cardiac functions. Based on GSE114695 profile data, REEP5 was decreased in the left ventricle of MI mice. However, its role and potential mechanism in MI remain to be investigated. In the present study, the mouse MI model was established by ligation of the left anterior descending artery. REEP5 expression was downregulated in the infarct penumbra area of MI mice. Next, its role during MI was explored by gain-of-function. Interestingly, REEP5 overexpression improved left ventricular function of mice with MI, accompanied with reduced infarct size. In cardiomyocytes, REEP5 overexpression inhibited ER stress, accompanied with repressive phosphorylation of PERK and IRE1α, and the decreased nuclear translocation of ATF6. Subsequently, REEP5 overexpression downregulated the levels of Chop and cleaved caspase-12, further alleviating ER stress-induced apoptosis, which was consistent with the in vivo results. Moreover, REEP5 was found to bind to C-type lectin member 5 A (CLEC5A), a protein that triggers cardiac dysfunction. CLEC5A, whose expression was elevated in hypoxia-induced cell models, led to cardiomyocyte apoptosis. Noteworthily, REEP5 overexpression markedly abolished the effects of CLEC5A on ER stress-induced apoptosis. Taken together, REEP5 mediated the function of CLEC5A to relieve MI via inhibiting ER stress-induced apoptosis in vivo and in vitro. REEP5 may be a promising target for treating MI.

**Supplementary Information:**

The online version contains supplementary material available at 10.1186/s12872-024-04018-3.

## Introduction

MI (myocardial infarction) is a serious manifestation of coronary artery disease [1]. MI happens when blood stops flowing normally to the heart, resulting in injured heart muscle due to lack of oxygen [2]. MI can induce the death of cardiomyocytes, therefore leading to cardiac dysfunction [3, 4]. The symptoms of MI are nausea, vomiting, chest pain, shortness of breath, abnormal heartbeat and anxiety [5]. MI has a substantial footprint on lifestyle and health of individuals due to its high morbidity and mortality [6]. MI treatment has focused on protecting the heart from heart failure. Moreover, intervention therapy and coronary artery bypass grafting are commonly used in clinical practice, which may effectively prevent the expansion of infarct area [3, 7]. Despite advances in MI treatment, developing new therapeutic targets remains a major research goal.

Receptor expression-enhancing proteins (REEPs) are an evolutionarily conserved family those are critical to the structure and function of endoplasmic reticulum (ER) [8]. REEP5 (also known as DP1) is a member of REEP family [9]. REEP5 deficiency deforms the sarcoplasmic reticulum (SR) structure, resulting in suppressed cardiac contractility [9]. Lee et al. also reported that REEP5 depletion led to cardiac dysfunction, compromised myocyte contractility and decreased cell viability [10]. Data from GSE114695 gene expression profile suggested that the expression of REEP5 was decreased in the left ventricle of MI mice. However, the role of REEP5 in MI is unclear.

One of the mechanisms that initiates MI is the stress response mediated by ER [11]. Oxidative stress, ischemic injury and altered expression of normal or folded defective proteins result in accumulation of unfolded proteins, which is known as ER stress [12]. Accumulating evidence suggests that ER stress is implicated in the pathogenesis of myocardial ischemia-reperfusion (MI/R) injury, myocardial hypertrophy, diabetic cardiomyopathy and other cardiovascular diseases [13–16]. ER stress is activated by three ER stress sensors, including PERK, IRE1α and ATF6 during MI, and it contributes to causing apoptosis and fibrosis [17–19]. Therefore, inhibition of ER stress is an important strategy to alleviate MI. Notably, REEP5 affects endoplasmic retinal structure and cargo carrying capacity, further regulating the intracellular transport system [8]. In addition, REEP5 depletion activated ER stress in cardiomyocytes [10]. However, whether REEP5 protects the heart from ER stress to attenuate MI needs to be further verified.

C-type lectin member 5 A CLEC5A (or MDL-1) is a common recognition receptor for some pathogenic microorganisms [20]. CLEC5A enhanced the inflammatory responses in myeloid cells and induced acute lung injury in pneumonia [21, 22]. Results of GSE114695 profile showed that CLEC5A was highly expressed in the cardiac tissues of MI mice. Additionally, CLEC5A knockdown prevented cardiac dysfunction by inhibiting macrophage polarization, NLRP3 inflammasome activation and pyroptosis [23]. However, the role of CLEC5A in MI is poorly understood. Of note, analysis using BIOGRID and HITPREDICT databases indicated that there was a possible binding between CLEC5A and REEP5, but whether REEP5 mediates the role of CLEC5A during MI requires further identification. Totally, the current study was aimed to explore the role and mechanism of CLEC5A and REEP5 during MI.

## Materials and methods

### DEGs identification from GSE114695 chip

Gene expression profile (GSE114695, https://www.ncbi.nlm.nih.gov/geo/query/acc.cgi?acc=GSE114695) was downloaded from the GEO database. Differently expressed genes (DEGs) from sham and MI samples were identified as |log2FC|>1 and *P* < 0.05. Heat map was drawn to show DEGs expression profile. The biological function and pathways involved in DEGs were analyzed with GO and KEGG enrichment. In addition, protein-protein-interation (PPI) network was performed using GENEMANIA database.

### Animal models of MI

The study protocol was approved by Ethical Medical Committee of Jinzhou Medical University and performed in accordance with the Guidelines for the Care and Use of Experimental Animals. Male C57BL/6 mice (aged 8 weeks) were used after 1 week of adaptation (temperature: 25 °C; humidity: 45–55%; 12 h light/dark cycle). The mice were randomly divided into 4 groups (sham group, MI group, MI + Ad-vector group and MI + Ad-oeREEP5 group). After 1 week of feeding under standard environmental conditions, the mouse MI model was established by ligation of the left anterior descending artery. A longitudinal incision about 1 cm long was made along the rib direction between the 3th and 4th ribs, centered on the strongest point of the left apex of the chest. Once inside the chest, the pericardium was cut to expose the left auricle, and the heart was gently pinched out of the void. The left anterior descending artery was ligated using a 5 − 0 suture. The suture was passed approximately 2 mm below the tip of the left auricle. In the sham group, only thoracotomy was performed without ligation. Successful ischemia was confirmed when the outer surface of the anterior wall of the left ventricle turned pale. After ligature, mice in MI + Ad-vector and MI + Ad-oeREEP5 were injected with Ad-vector or Ad-oeREEP5 (10^9^ pfu/ml, 20 µl injection) at 3 points outside the infarct area. While mice in control and MI were group received the same amount of PBS. Left ventricular functions (left ventricular end systolic diameter (LVESD), left ventricular end-systolic volume (LVESV), left ventricular end diastolic dimension (LVEDD), left ventricular end-diastolic volume (LVEDV), EF (= LVEDV-LVESV/LVESV×100%) and FS (= LVEDD-LVESD/LVESD×100%)) of mice were determined with echocardiography at day 7 after injection. At the end of the echocardiography, the rats were sacrificed by CO_2_ asphyxia, and myocardial tissues were harvested for subsequent experiments.

### Cell treatment and transfection

AC16 cells were obtained from the iCell (Shanghai, China) and cultured in DMEM/F12 (Biosharp, Hefei, China) supplementing 10% fetal bovine serum (FBS, Tianhang Biotechnology, China). Cells were seeded in 6-well plates at 4 × 10^5^ cells/well and cultured at 37 °C and 5% CO_2_. For hypoxia treatment, the cells were seeded in a 6-well plate (4 × 10^5^cells/well) and incubated in a normoxia or hypoxia (1% O_2_, 5% CO_2_ and 94% N_2_) condition at 37 °C for 24 h.

For cell transfection, REEP5 CDS or CLEC5A CDS were synthesized by General bio (Anhui, China), inserted into pShuttle-CMV vector (Fenghui Biotechnology, Changsha, China) and transfected into AC16 cells using Lipofectamine 3000 (Invitrogen, USA) according to the manufacturer’s potocol. After transfection for 24 h, cells were incubated at 37℃ under a hypoxia condition for 24 h and subsequently collected for further analysis.

### TUNEL staining

Myocardial tissues were embedded in paraffin and cut into 5 μm-thick sections. The sections were dewaxed in xylene, hydrated in anhydrous ethanol (95%-85%-75%), and then added with Triton X-100 (0.1% sodium citrate salt configuration, 200 µl, Beyotime, Shanghai, China) and treated for 15 min. TUNEL reaction solution (200 µl) was added to the cells and incubated at 37℃ for 1 h in the dark. The nuclei were counterstained with DAPI (Aladdin Biochemical Technology, Shanghai, China) for 5 min, and images were captured with a fluorescence microscope (Olympus, Japan).

### Triphenyl tetrazolium chloride (TTC) staining

TTC staining was used to detect left ventricular myocardial infarction area. The frozen heart was into five pieces and placed in a petri dish. Sections were then stained with 1% TTC solution at 37℃ for 10 min away from light. The heart was turned over and stained at 37℃ for additional 10 min. The staining was captured, and the percentage of infarct area (infarct weights/whole heart weights ×100%) was calculated using ipp6.0 software (Media cybernetics, USA).

### Immunohistochemical (IHC) staining

Myocardial tissues were embedded in paraffin and sliced into 5 μm-thick sections. Sections were dewaxed in xylene and hydrated in ethyl alcohol (95%-85%-75%). Then the sections were placed in antigen repair solution and continuously heated for 10 min. After the heating was stopped, the sections were taken out and soaked in PBS for 5 min. Subsequently, the sections were treated with 3% H_2_O_2_ to eliminate the endogenous peroxidase and blocked with 1% BSA for 15 min. Then the sections were incubated with primary antibody REEP5 (1:100, Affinity, Changzhou, China) at 4 °C overnight, followed by incubation with the goat anti-rabbit IgG (1:500, ThermoFisher, USA) for 1 h at 37 °C. Next, the sections were stained with DAB and counterstained with hematoxylin. The staining was observed by a microscope.

### Immunofluorescence (IF) staining

Cells were fixed with 4% paraformaldehyde for 15 min and permeabilized using 0.1% Triton X-100 for 30 min. Then 1% BSA was added and incubated at room temperature for 15 min. Primary antibody (ATF6, 1:100, Affinity, Changzhou, China) were added and incubated overnight at 4 °C, followed by incubation with Cy3-conjugated goat anti-rabbit IgG (1:200, Invitrogen, USA) for 1 h at room temperature in the dark. The sections were counterstained with DAPI, and images were obtained under a microscope.

### Co-immunoprecipitation (co-IP)

The protein was extracted and quantified by a BCA protein concentration detection kit (Proteintech, Wuhan, China). According to the instructions of the immunoprecipitation test kit, 20 µl AminoLink conjugated resin was added to Pierce centrifugal column, and 200 µl cross-linking buffer was used to wash resin. The target IP antibody was added to 20× crosslinked buffer and the volume was adjusted to 200 µl. After centrifuging 200 µl of 1× crosslinked buffer, the lysate was separately added to the corresponding resin that has solidified the corresponding antibody and oscillated for 2 h. After centrifugation at room temperature, the column was placed in a new collection tube, 200 µl IP washing buffer was added and centrifuged. Then the centrifuge column was placed in a collection tube and 50 µl elution buffer was added. After standing at room temperature for 5 min and centrifugation, samples were applied to western blot. The information of antibodies detected were as follows: REEP5 antibody (1: 1000, Affinity) and CLEC5A antibody (1:1000, Abcam, UK).

### Reverse transcription-PCR (RT-PCR) and qPCR

TRIpure and chloroform were employed to extract total RNA. Briefly, 1 ml TRIpure lysate, followed by 200 µl chloroform was added to the sample. After centrifugation at 4℃ for 10 min, the aqueous phase was transferred to a new centrifuge tube and isopropyl alcohol was added. After mixing, the aqueous phase was placed at -20℃ overnight. The sample was added with 1 ml 75% ethanol, centrifuged at 4℃ for 3 min, and the supernatant was discarded. The concentration of RNA in each sample was determined using an ultraviolet spectrophotometer NANO 2000 (ThermoFisher, Scientific, USA). Reverse transcriptase (BeyoRT II M-MLV, Beyotime, China) was used to synthesize cDNA. The PCR products were detected by electrophoresis (1.5% agarose gel) and photographed under the gel imaging system. In addition, qPCR amplification was conducted using a SYBR green kit (Solarbio, Beijing, China) and analyzed using an ExicyclerTM96 PCR reaction system (Bioneer Corporation, Korea). β-actin served as the internal control, and the relative mRNA levels were detected using the 2^−△△CT^ method.

The primers for RT-PCR were provided as follows:

XBP1s: F: 5’- GAATGAAGTGAGGCCAGTGG − 3’;

XBP1u: F: 5’- ACTGGGTCCTTCTGGGTAGA − 3’.

The primers for qPCR were provided as follows:

REEP5: F: 5’- CGCATCATCCGTCCTTT − 3’;

REEP5: R: 5’- TTTCTTCGCTTCTTTAGTG − 3’.

### Western blot

Tissues or cells were lysed using RIPA lysis buffer containing 1 mmol/l PMSF. After centrifugation at 4 °C for 3 min, the supernatant was collected as protein extract. The protein concentration was detected with a BCA protein assay kit (Beyotime). The protein sample was diluted with 5×Loading Buffer and boiled in water bath for 5 min to prepare the loading solution. Protein samples (30 µg) were separated by 10% SDS-PAGE and then transferred to PVDF membranes (ThermoFisher). After being blocked with 5% BSA for 1 h, the blots were incubated with the primary antibodies at 4 °C overnight. After washing with TBST for 5 min, the blots were incubated with the secondary antibodies (goat anti-rabbit IgG, 1: 10,000, Proteintech or goat anti-mouse IgG, 1: 10,000, Proteintech) at 37 °C for 40 min. The membranes were then incubated with ECL (7 Sea biotech, Shanghai, China) and quantified by Gel-Pro-Analyzer.

The primary antibodies used were as follows: REEP5 antibody (1: 1000, Affinity); CLEC5A antibody (1: 1000, Bioss, Beijing, China); GRP78 antibody (1: 1000, ABclonal, Wuhan, China); PERK antibody (1: 1000, Affinity); p-PERK antibody (1: 1000, Affinity); IRE1α antibody (1: 1000, Affinity); p-IRE1α antibody (1: 1000, Affinity); ATF6 antibody (1: 1000, Affinity); Chop antibody (1: 1000, Affinity) and cleaved caspase-12 antibody (1: 1000, Affinity).

### Statistical analysis

Data were analyzed by GraphPad software and expressed as mean ± SD. P values < 0.05 were considered as statistically significant. Statistical differences between two groups were compared using a t-test. Statistical differences from multiple groups were performed using one-way analysis of variance, followed by Tukey test.

## Results

### Expression of DEGs in MI from GSE114695 profile

**Fig. 1:**
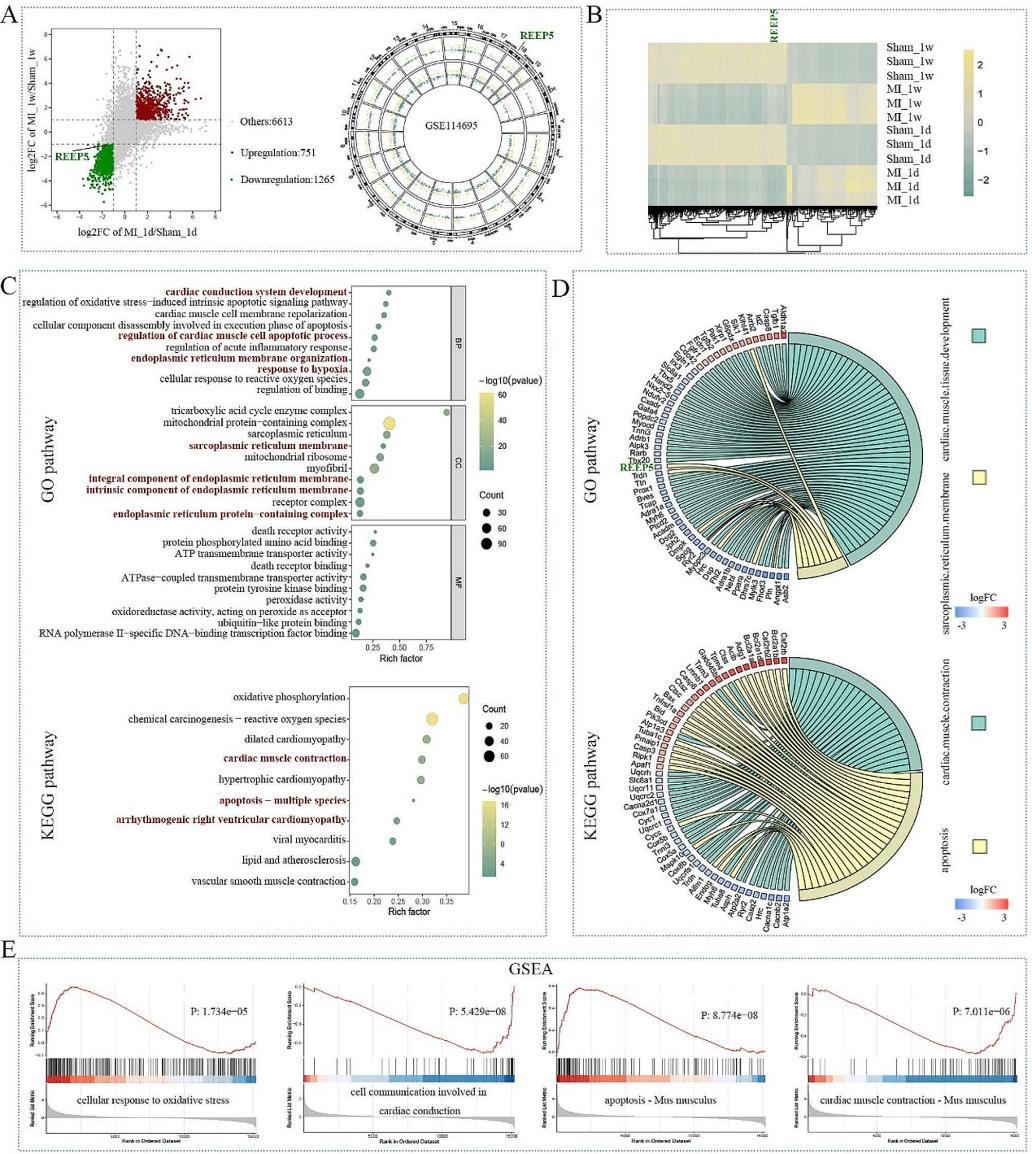
Expression of DEGs in MI from GSE114695 profile. (**A**) Volcano plot and Track of DEGs. (**B**) The expression heatmap of DEGs. (**C**) GO and KEGG enrichment analyzes of DEGs. (**D**) Chordal graph of DEGs enriched in several pathways. (**E**) GSEA plots of DEGs

We download GSE114695 profile and selected 1 day and 1 week data from MI and sham samples for bioinformatics analysis. Volcano plot showed gene expression and track suggested the chromosomal distribution of DEGs (Fig. 1A). There were 751 upregulated DEGs and 1265 downregulated DEGs in MI mice compared with sham mice. The expression heatmap of DEGs was also presented in Fig. 1B. To better understand the function of DEGs, GO enrichment was analyzed. As shown in Fig. 1C, the DEGs were classified into three functional groups. BP group results displayed that these DEGs were enriched in cardiac conduction system development, regulation of cardiac muscle cell apoptotic process, endoplasmic reticulum membrane organization, response to hypoxia and cellular response to reactive oxygen species. These data indicated that the DEGs were associated with the cardiac function, apoptotic signaling pathway, endoplasmic reticulum membrane organization and response to hypoxia. CC group analysis showed that the DEGs were mainly enriched in sarcoplasmic reticulum membrane, integral component of endoplasmic reticulum membrane, intrinsic component of endoplasmic reticulum membrane and endoplasmic reticulum protein-containing complex. These findings implied that the DEGs were related with the sarcoplasmic reticulum membrane and endoplasmic reticulum membrane. MF group data indicated that the DEGs were enriched in death receptor activity. Moreover, for KEGG evaluation, the DEGs were involved in dilated cardiomyopathy, apoptosis and arrhythmogenic right ventricular cardiomyopathy (Fig. 1C). We also presented the DEGs enriched in several pathways during GO and KEGG analyzes, as shown in chordal graphs and GSEA (Fig. 1D-E).

In addition, BP group analysis showed that REEP5 was enriched in sarcoplasmic reticulum membrane. (Fig. 1C). REEPs are critical to the structure and function of endoplasmic reticulum [8] However, the effect of REEP5 on endoplasmic reticulum stress during MI remains unknown. Based on the above results, REEP5 was selected as the candidate factor, and its function was subsequently explored in this work.

### REEP5 overexpression alleviates hypoxic-induced cardiomyocyte injury

**Fig. 2:**
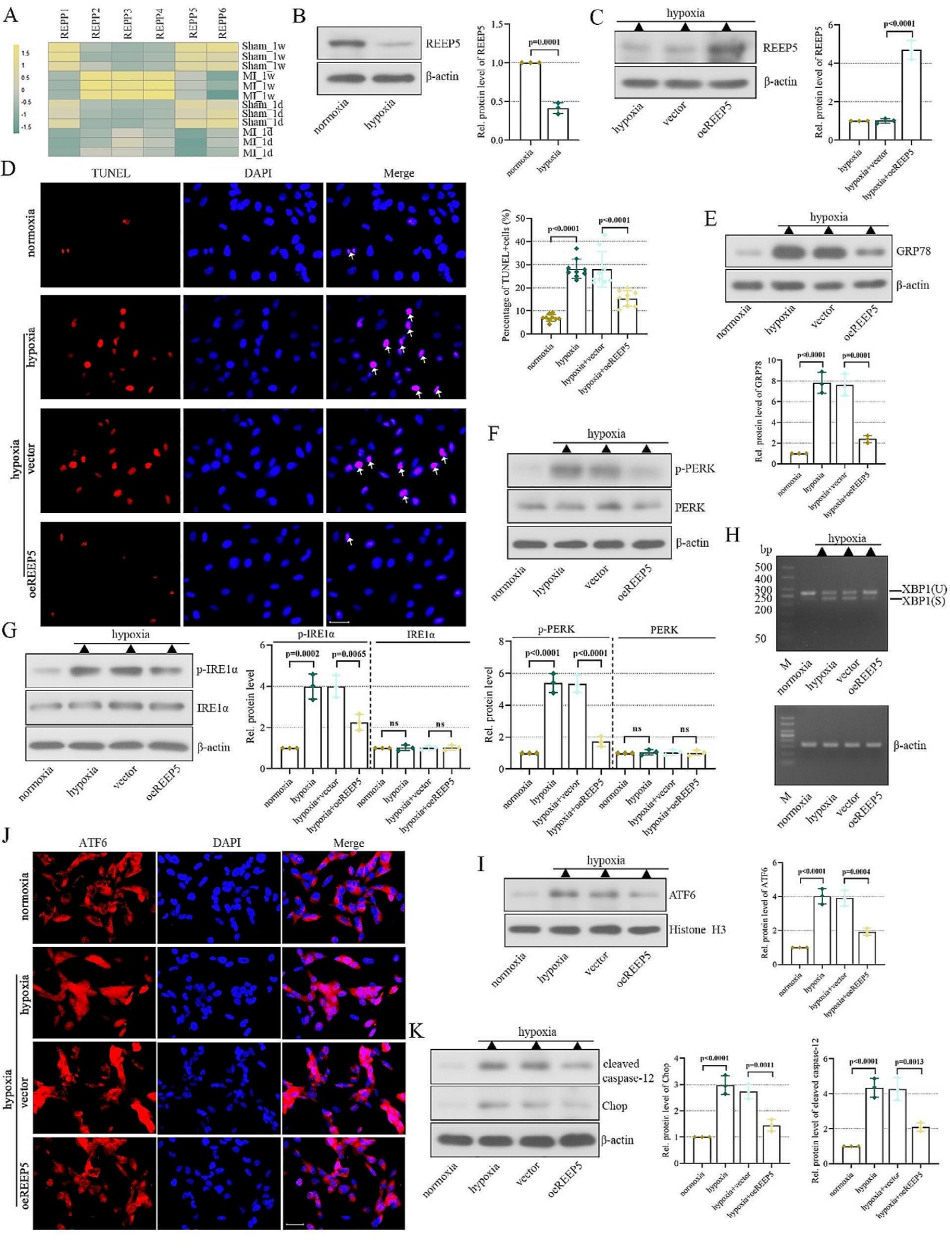
REEP5 overexpression alleviates hypoxic-induced cardiomyocyte injury. (**A**) Heat map showed the expression of REEP family members from GSE114695. (**B**) The expression of REEP5 was detected by western blot. (**C**) The overexpression efficiency of REEP5. (**D**) Apoptosis was detected by TUNEL staining (scale bar = 50 μm, the arrows represent TUNEL-positive cells). (**E**) GRP78 levels in AC16 cells. (**F**-**G**) PERK, P-PERK, IRE1α and P-IRE1α levels in AC16 cells. (**H**) XBP1 levels in AC16 cells were detected by RT-PCR. (**I**-**J**) ATF6 levels in nuclear. (**K**) Chop and cleaved caspase-12 levels in AC16 cells. (*P* < 0.01, *P* < 0.001, *P* < 0.0001), *n* = 3

The expression of REEPs was presented in Fig. 2A. It was shown that REEP5 was markedly downregulated in the left ventricle of MI mice. To elucidate the expression of REEP5 in vitro, we established a hypoxic cell model and found that its expression was significantly reduced under hypoxia conditions (Fig. 2B). Next, we explored the role of REEP5 through gain-of-function. The overexpression efficiency of REEP5 was detected by western blot after transfection of REEP5 overexpression plasmid for 24 h and hypoxia culture for 24 h (Fig. 2C). Then we assessed the impact of REEP5 overexpression on cardiomyocyte apoptosis using TUNEL assay. As presented in Fig. 2D, hypoxia significantly increased the number of TUNEL-positive cells, which was abolished by REEP5 overexpression.

ER stress exerts a key role in cardiac diseases via apoptosis induction [24]. Therefore, we sought evidence on whether REEP5 suppressed the ER stress response. As illustrated by western blot analysis, hypoxia led to the exacerbation of ER stress, along with increased GRP78 expression, PERK and IRE1α phosphorylation, elevated the nuclear translocation of ATF6 and active spliced XBP-1 (XBP-1s) levels, whereas, the aggressive ER stress was mitigated by REEP5 overexpression (Fig. 2E-I). Subsequently, IF staining results showed that REEP5 overexpression inhibited the nuclear translocation of ATF6 (Fig. 2J).

To ascertain the potential role of REEP5 in ER stress-associated apoptosis, the levels of proapoptotic factors were examined. As demonstrated in Fig. 2K, REEP5 overexpression decreased the levels of cleaved caspase-12 and Chop, thus reversing the effect of hypoxia on these protein levels. Collectively, these results indicated that upregulation of REEP5 suppressed ER stress-associated apoptosis.

### REEP5 overexpression attenuates MI of mice

**Fig. 3:**
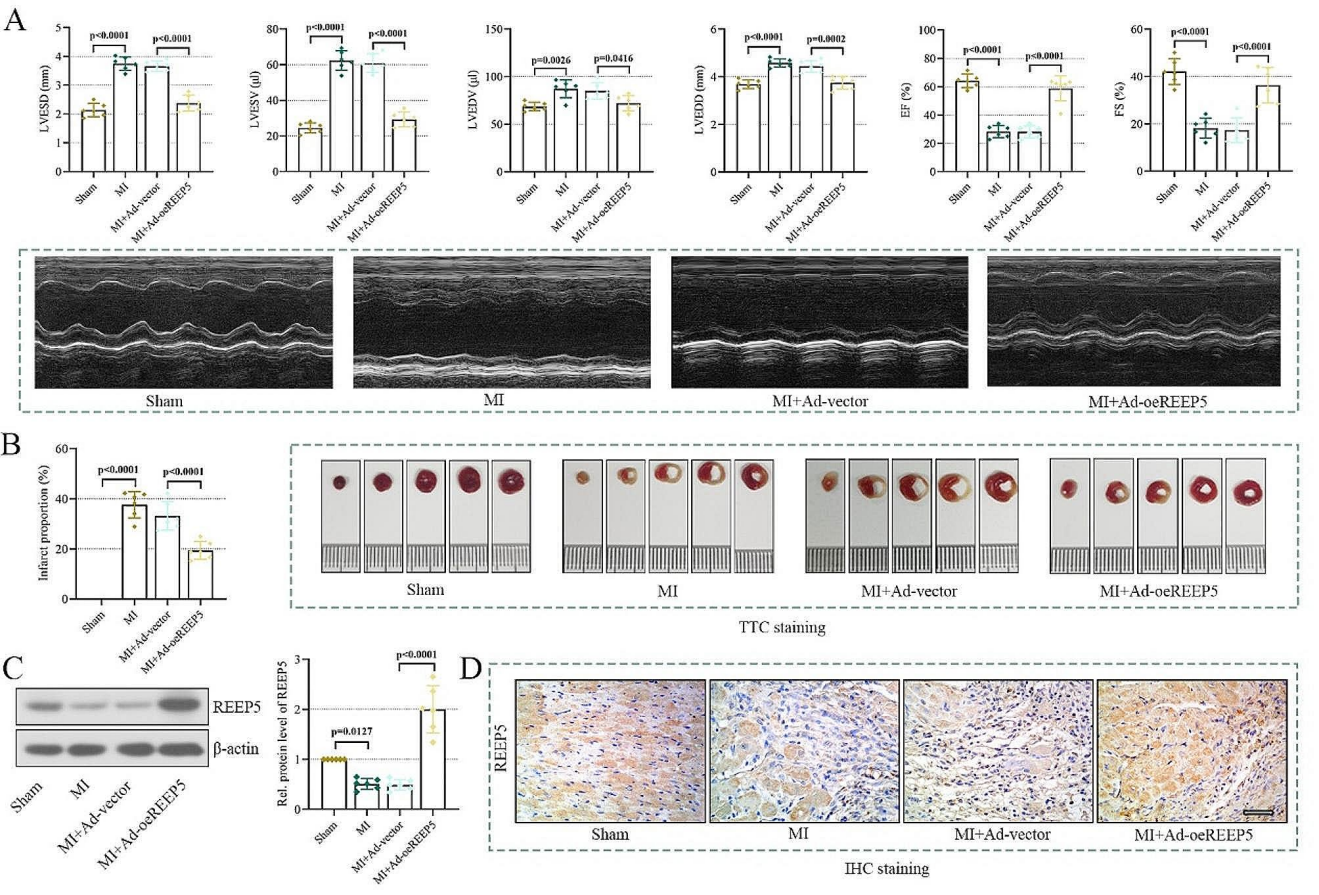
REEP5 overexpression attenuates MI of mice. (**A**) LVEDD, LVESV, LVESD, LVEDV, EF and FS were measured, and representative echocardiograms were displayed. (**B**) TTC staining was used to detect left ventricular myocardial infarction area, and infarct proportion was calculated. (**C**-**D**) The expression of REEP5 in infarct penumbra was detected by western blot and IHC staining (scale bar = 50 μm). (*P* < 0.05, *P* < 0.01, *P* < 0.001, *P* < 0.0001), *n* = 6

To further certify the protective effect of REEP5 in vivo, we established a MI mouse model by ligation of the left anterior descending artery. As displayed in Fig. 3A, MI mice exhibited greatly upregulated levels of LVESD, LVESV, LVEDD and LVEDV compared with the sham group, indicating impaired cardiac systolic function. Moreover, EF and FS were markedly downregulated in the MI group, which also potentiated the damage of cardiac function. However, REEP5 overexpression mice showed the opposite alterations, implying that REEP5 restrained myocardial dysfunction of MI mice. The above protective effect of REEP5 overexpression was further supported by the results of TTC staining. As expected, the MI mice showed a notable MI injury with an extend infarct area and increased infarct proportion, while REEP5 overexpression remarkably abrogated these alterations (Fig. 3B). Then we measured the expression of REEP5 using western blot and IHC staining. As reflected in Fig. 3C-D, REEP5 levels were obviously decreased in the infarct penumbra area of MI mice, but was increased after infection with REEP5 overexpression. These observations provided evidence that REEP5 protected mice from MI-triggered cardiac injury.

### REEP5 overexpression inhibits ER stress-induced apoptosis

**Fig. 4:**
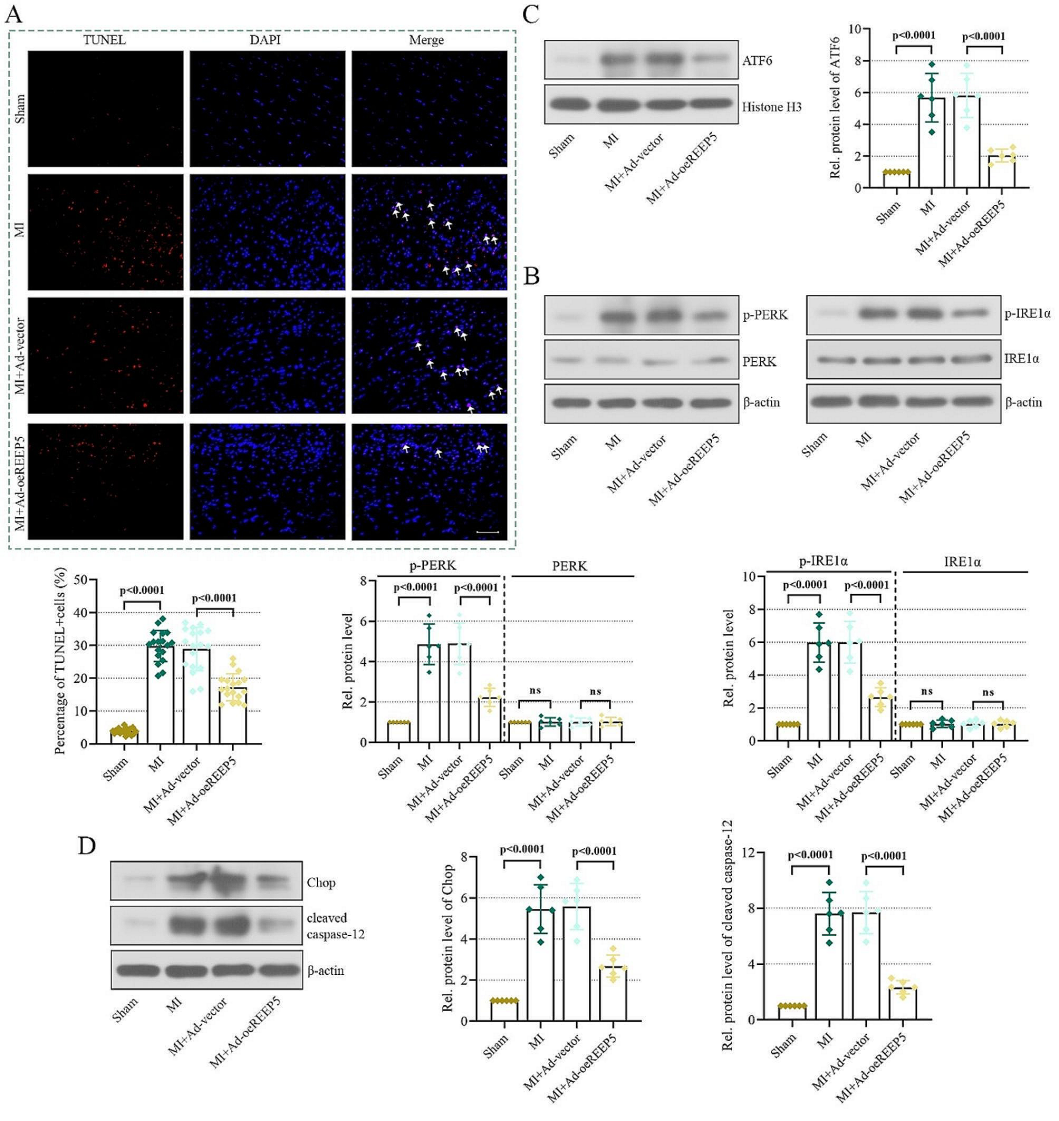
REEP5 overexpression inhibits ER stress-induced apoptosis. (**A**) Apoptosis was detected by TUNEL staining (scale bar = 50 μm, the arrows represent TUNEL-positive cells). (**B**) PERK, P-PERK, IRE1α and P-IRE1α levels. (**C**) ATF6 levels in nuclear. (**D**) Chop and cleaved caspase-12 levels. (*P* < 0.0001), *n* = 6

To further verify the relieving effect of REEP5 on ER stress-associated apoptosis in vivo, we also detected apoptosis and the levels of three branches that activate ER stress. As suggested in Fig. 4A, TUNEL-positive cells were substantially increased in the MI group and decreased in REEP5 overexpression mice. Furthermore, during MI, elevated PERK and IRE1α phosphorylation and the nuclear translocation of ATF6 activated ER stress (Fig. 4B-C). Whereas, overexpression of REEP5 decreased the levels of these protein, further attenuating severe ER stress. Subsequently, REEP5 overexpression repressed the levels of Chop and cleaved caspase-12, also negating the apoptosis induced by ER stress (Fig. 4D). Based on the above results, it was demonstrated that REEP5 overexpression inhibited ER stress-associated apoptosis, therefore alleviating MI.

### CLEC5A aggravates hypoxic-induced cardiomyocyte injury and downregulates REEP5 levels

**Fig. 5:**
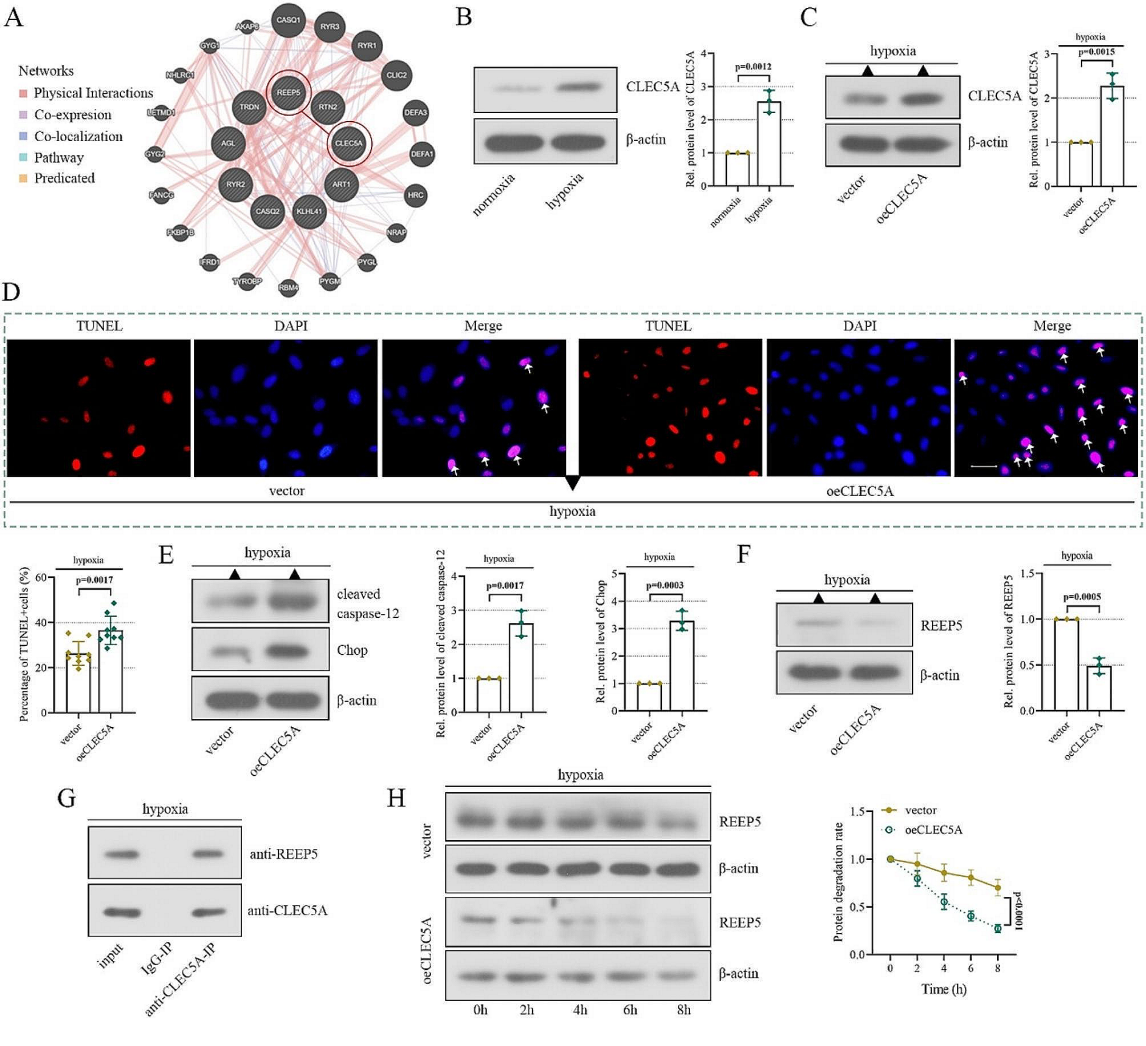
CLEC5A aggravates hypoxic-induced cardiomyocyte injury and downregulates REEP5 levels. (**A**) The PPI network of DEGs. (**B**) The expression of CLEC5A in AC16 cells was detected by western blot. (**C**) The overexpression efficiency of CLEC5A. (**D**) Apoptosis was detected by TUNEL staining (scale bar = 50 μm, the arrows represent TUNEL-positive cells). (**E**) Chop and cleaved caspase-12 levels in AC16 cells. (**F**) The protein expression of REEP5 in CLEC5A-overexpressed cells. (**G**) CLEC5A binding to REEP5 was detected by co-IP. (**H**) After CHX treatment for 0, 2, 4, 6 and 8 h, the expression of REEP5 was detected by western blot, and the degradation rate of REEP5 protein was calculated. (*P* < 0.01, *P* < 0.0001), *n* = 3

It has been reported that CLEC5A is significantly upregulated in the left ventricle of MI mice, and knockdown of CLEC5A ameliorates cardiac dysfunction [23]. In addition, online database data showed that there was a potential binding between CLEC5A and REEP5 (Fig. 5A). Therefore, we analyzed the potential interaction between CLEC5A and REEP5 (Fig. 5A). Accordingly, we next explored the mechanism of CLEC5A and REEP5 during MI. Herein, western blot analysis was applied to determine the expression of CLEC5A. Elevated CLEC5A levels were observed under hypoxia conditions (Fig. 5B). Then we overexpressed CLEC5A in cardiomyocytes using plasmid transfection, whose transfection efficiency was verified by western blot (Fig. 5C). We further explored the role of CLEC5A in cell apoptosis. The results of TUNEL staining and western blot confirmed that CLEC5A overexpression increased TUNEL-positive cells, as well as the levels of Chop and cleaved caspase-12 (Fig. 5D-E), implying that CLEC5A aggravated hypoxia-induced cardiomyocyte injury. Strikingly, we found that the expression levels of REEP5 was decreased in cells overexpressing CLEC5A (Fig. 5F and Supplementary Fig. 1), indicating that there may be a possible modulation of CLEC5A on REEP5. To verify this hypothesis, a co-IP experiment was conducted. As indicated by the results of co-IP, CLEC5A bound with REEP5 (Fig. 5G). Furthermore, in cells with CLEC5A overexpression, the protein degradation of REEP5 was much quicker than that in control cells, indicating that CLEC5A promoted REEP5 protein degradation (Fig. 5H). These findings suggested that CLEC5A not only bound to REEP5, but also promoted its protein degradation.

### REEP5 mediates the function of CLEC5A in cardiomyocytes

**Fig. 6:**
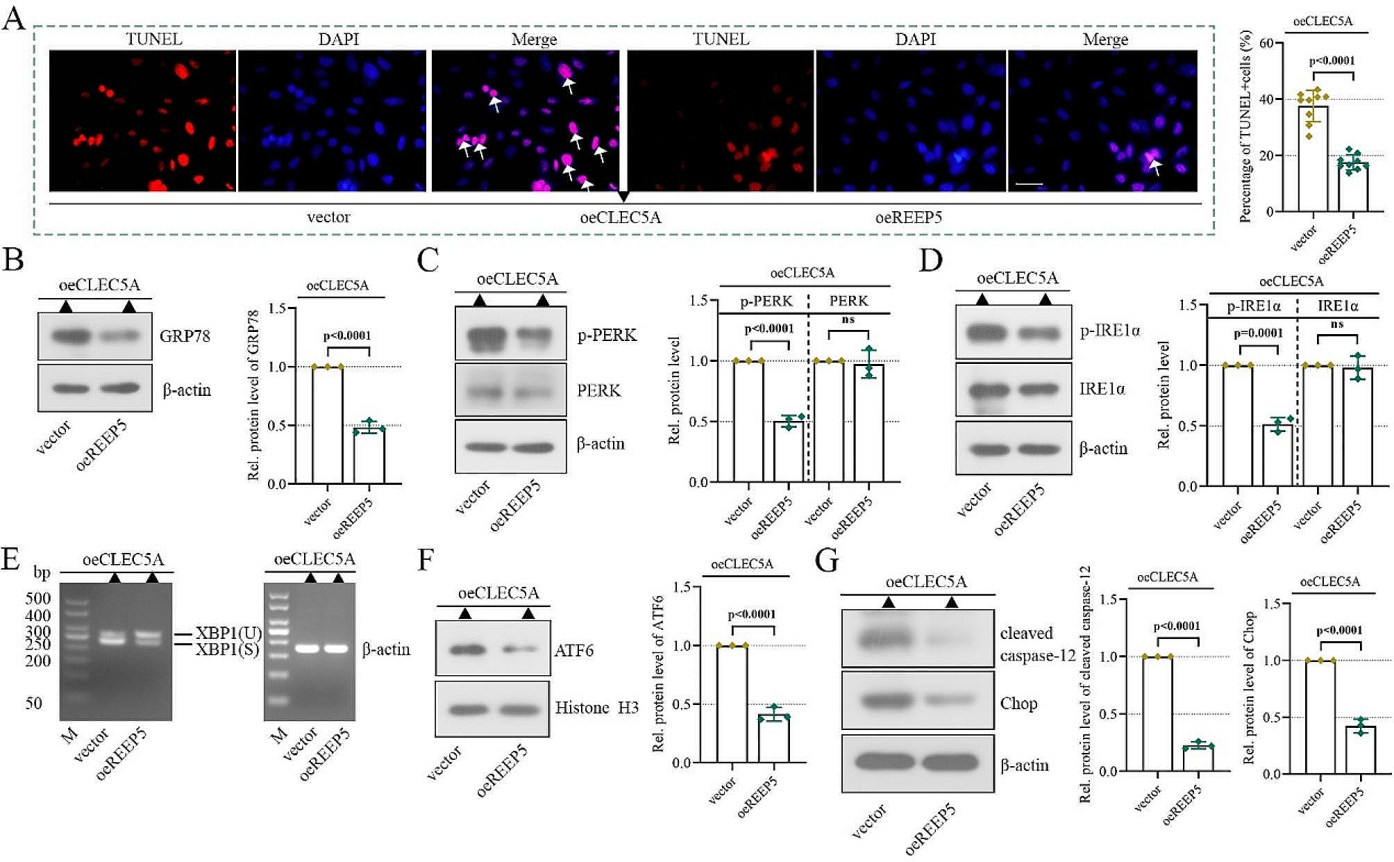
REEP5 mediates the function of CLEC5A in cardiomyocytes. (**A**) Apoptosis was detected by TUNEL staining (scale bar = 50 μm, the arrows represent TUNEL-positive cells). (**B**-**E**) GRP78, PERK, P-PERK, IRE1α, P-IRE1α and XBP1 levels. (**F**) ATF6 levels in nuclear. (**G**) Chop and cleaved caspase-12 levels. (*P* < 0.001, *P* < 0.0001), *n* = 3

To appraise whether REEP5 mediates the function of CLEC5A during MI, cardiomyocytes were co-transfected with CLEC5A overexpression and REEP5 overexpression plasmids and subsequently cultured under a hypoxia condition for 24 h. Our data illustrated that the TUNEL-positive cells, levels of GRP78, PERK and IRE1α phosphorylation and XBP-1s were all increased in CLEC5A overexpressed cells, while REEP5 overexpression decreased them (Fig. 6A-E). In addition, CLEC5A overexpression elevated the nuclear translocation of ATF6 and the levels of Chop and cleaved caspase-12, while these alterations were negated by REEP5 overexpression (Fig. 6F-G). In summary, these data suggested that REEP5 mediated the function of CLEC5A during MI.

## Discussions

In this work, we explored the potential role of REEP5 during the pathogenesis of MI. Our findings identified that REEP5 was decreased in the infarct penumbra area of MI mice. Its overexpression protected myocardial function via inhibiting ER stress-induced apoptosis in vivo and vitro. Besides, the mechanism that REEP5 mediated the role of CLEC5A during MI was revealed. It was demonstrated that CLEC5A bound with REEP5 and promoted REEP5 protein degradation. REEP5 mediated the function of CLEC5A on ER stress-associated apoptosis. Our findings verified that REEP5 may be a novel potential target for MI treatment.

**Fig. 7:**
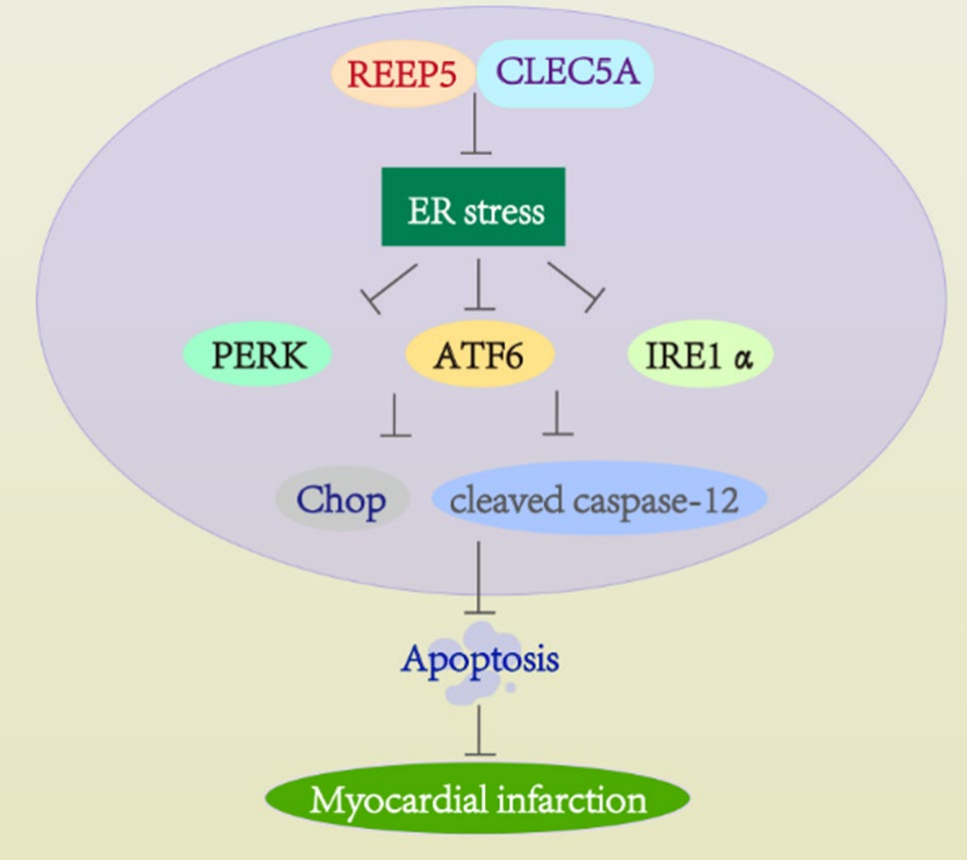
Diagram of the role and potential mechanism of REEP5 during MI. REEP5 mediated the function of CLEC5A to alleviate MI via inhibiting ER stress-induced apoptosis

Growing evidence indicated that REEP5 expression was decreased in the failing hearts [9, 10]. In the present study, we analyzed gene expression profile in MI mice (GSE114695) and found that REEP5 expression was also decreased in the left ventricle of MI mice. Herein, we explored the impact of REEP5 on MI-induced myocardial dysfunction in vivo. As displayed, the MI mice exhibited impaired cardiac dysfunction, while REEP5 overexpression mice showed the protective alterations, suggesting that REEP5 protected mice from MI injury, which was consistent with a previous report that REEP5 depletion leads to impaired cardiac contractility and cardiac dysfunction [9]. Thus, we hypothesized that REEP5 may contributed to the cardiac dysfunction during MI, and that rectification of REEP5 expression may benefit to the treatment of MI.

The ER is a lipid bilayer extension of the outer nuclear membrane, consisting of continuous peripheral ER tubules and dispersed membrane layers [25]. The ER/SR is a multifunctional organelle responsible for many fundamental cellular processes in eukaryotic cells, including protein translation, lipid synthesis, Ca^2+^ cycling and protein transport [26, 27]. The members of REEP family contain the reticular homeodomain (RHD), which is critical for inducing and stabilizing high membrane curvature of endoplasmic reticulum tubule cross Sect. [28]. REEP5 is a conserved heart-rich membrane protein whose C-terminal domain contributes to the integrity of the ER. REEP5 has been reported to regulate intracellular transport through affecting ER structure and cargo carrying capacity [28]. The ischemic and hypoxic-damaged hearts often experience oxidative stress and inflammation, all of which disrupt the ER, resulting in ER stress [29]. ER stress is activated by three branches (PERK, IRE1α and ATF6) and it is involved in the pathogenesis of MI [30–33]. Notably, Luo et al. demonstrated that MI induced ER stress and provoked cardiac apoptosis and fibrosis [34]. Previous studies also revealed that REEP5 served as an SR membrane sculptor to modulate cardiac function. Its deficiency activates ER stress in cardiomyocytes, leading to SR/ER membrane instability and intracavity vacuolation, along with reduced contractility of cardiomyocytes and disrupted Ca^2+^ cycling [10, 35]. REEP5 interacts with several members of the reticulin and adnexin protein families, as well as cytoskeleton-associated protein 4. These interacting proteins have previously been associated with ER formation functions, including high curvature formation, tubule fusion, and intracavitary spacing [35]. Thus, we then explored the involvement of REEP5 in ER stress-associated apoptosis during MI. In vitro data suggested that hypoxia exacerbated ER stress, as evidenced by increased PERK and IRE1α phosphorylation and nuclear ATF6 levels, thus leading to the upregulation of GRP78 (the master of the unfolded protein response) expression and active XBP-1 splicing [36]. Whereas, these phenotypic alterations were abrogated by REEP5 overexpression, indicating that REEP5 suppressed hypoxia-simulated ER stress. Also, we found that REEP5 overexpression suppressed apoptosis, as reflected by decreased levels of Chop and cleaved caspase-12 (symbols of apoptosis) [37], confirming a protective role of REEP5 on cardiomyocytes. The same results were also obtained from a mouse MI model. Moreover, there are many other risk factors during MI, such as oxidative stress, pyroptosis and inflammation [38–40]. Whether REEP5 affects other signals to relieve MI needs to be further explored.

Notably, data of online database showed that there was a potential binding between CLEC5A and REEP5. CLEC5A is a common recognition receptor and mediated inflammatory response in various disease [20–22]. Importantly, accumulating evidence has suggested that CLEC5A is a leading cause of MI, and CLEC5A knockdown suppresses the activation of NLRP3 inflammasome and pyroptosis in primary cardiomyocytes [23, 41]. Interestingly, Cheng et al. suggested that after ER stress, PERK promoted NRF2-mediated transcriptional activation of CLEC5A, thereby increasing CLEC5A expression [42]. These results indicated that CLEC5A might be implicated in the progression of MI. In this study, we certified that CLEC5A protein expression was increased in cardiomyocytes under a hypoxia condition, and CLEC5A overexpression enhanced cell apoptosis, thus aggravating hypoxia-induced cardiomyocyte injury. Accordingly, we further identified the mechanism of CLEC5A and REEP5. It was found that CLEC5A overexpression decreased REEP5 protein levels. Subsequently, our results demonstrated that CLEC5A bound with REEP5 and promoted its protein degradation. In addition, our data showed that CLEC5A decreased REEP5 mRNA levels, implying that CLEC5A may also regulate REEP5 transcription through other ways. Huang et al. indicated that CLEC5A regulated nuclear translocation of transcription factor NFATC1 [43]. In addition, JASPAR prediction showed that NFATC1 has potential binding sites in the promoter sequence of REEP5. Thus, we speculated that CLEC5A may influence the transcription of REEP5 by regulating some transcription factors, such as NFATC1. However, how CLEC5A affects REEP5 transcription remains to be explored in the future studies. Remarkably, CLEC5A overexpression promoted ER stress-associated apoptosis, while REEP5 overexpression abolished the effect of CLEC5A overexpression. These results implied that REEP5 mediated the function of CLEC5A during MI.

In summary, our findings suggest that REEP5 mediates the function of CLEC5A to alleviate MI by inhibiting ER stress-induced apoptosis (Fig. 7). These results provide new insights into the progression of MI. REEP5 may be a valuable target for MI.

### Electronic supplementary material

Below is the link to the electronic supplementary material.

Supplementary Material 1

Supplementary Material 2

## Data Availability

The datasets generated or analyzed during the current study are available from the corresponding author on reasonable request.
