# Supplementary figures and images for: REEP5 mediates the function of CLEC5A to alleviate myocardial infarction by inhibiting endoplasmic reticulum stress-induced apoptosis
Source: BMC Cardiovasc Disord. 2024 Jul 23;24:382. doi: 10.1186/s12872-024-04018-3 (PMC11265427; doi:10.1186/s12872-024-04018-3)

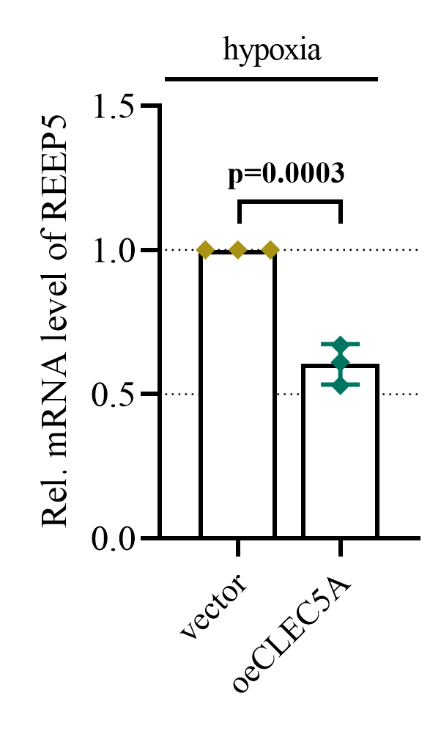


Supplementary Figure 1. The mRNA expression of REEP5 in CLEC5A-overexpressed cells. (P<0.001), n=3.

Supplement: Supplementary file 1 — Supplementary Material 1 [file 12872_2024_4018_MOESM1_ESM.docx]

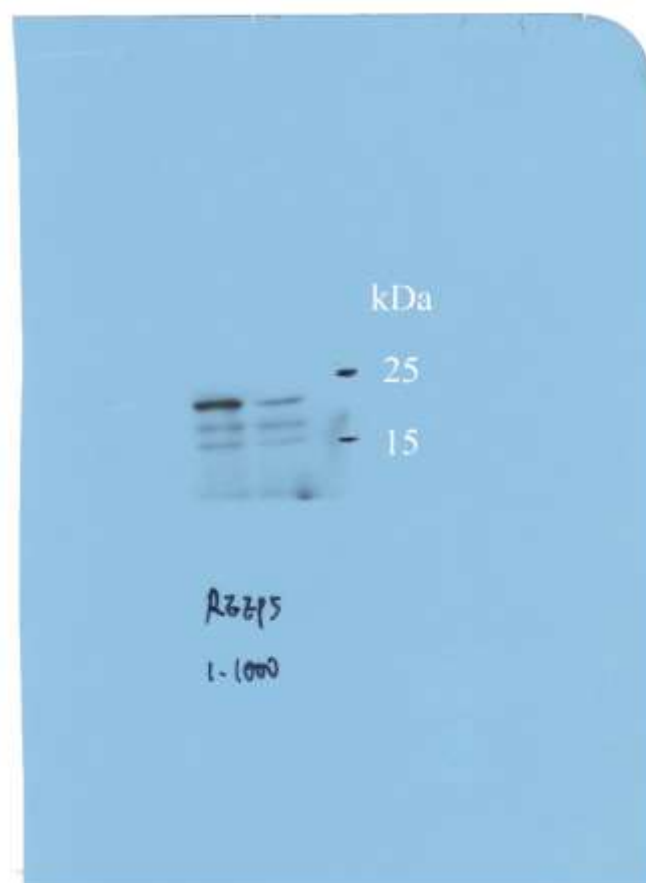

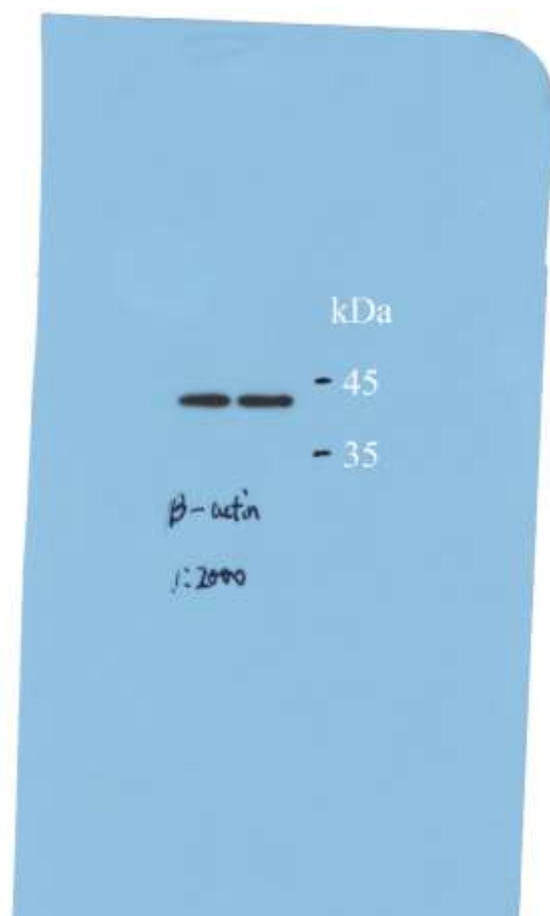

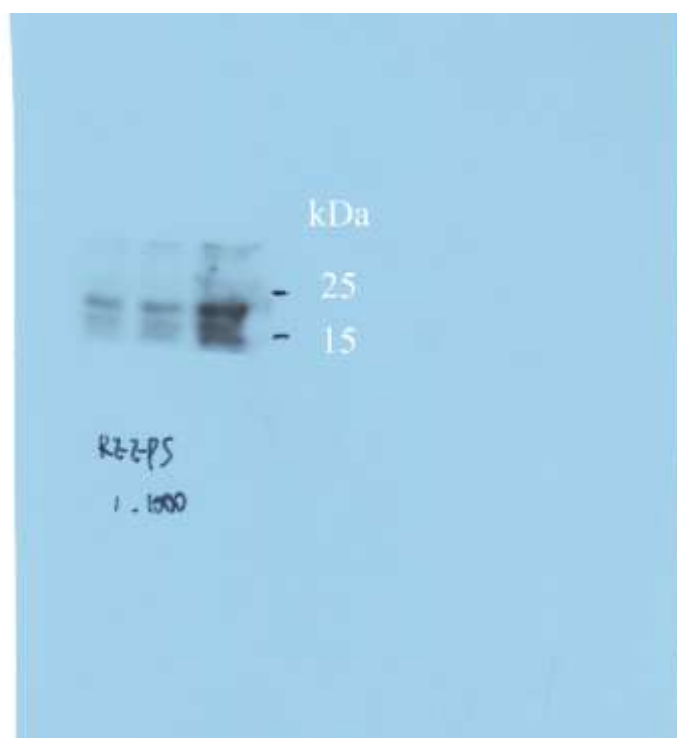

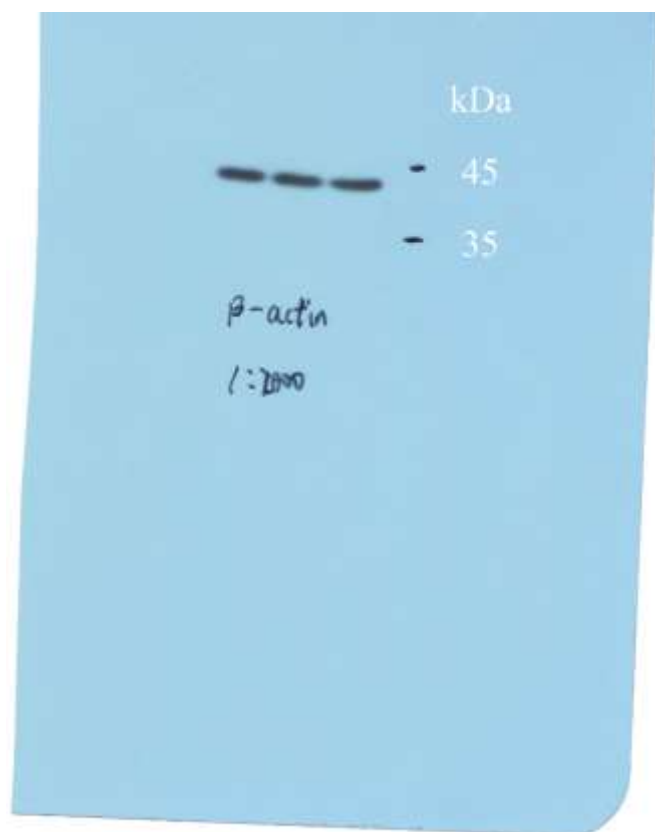

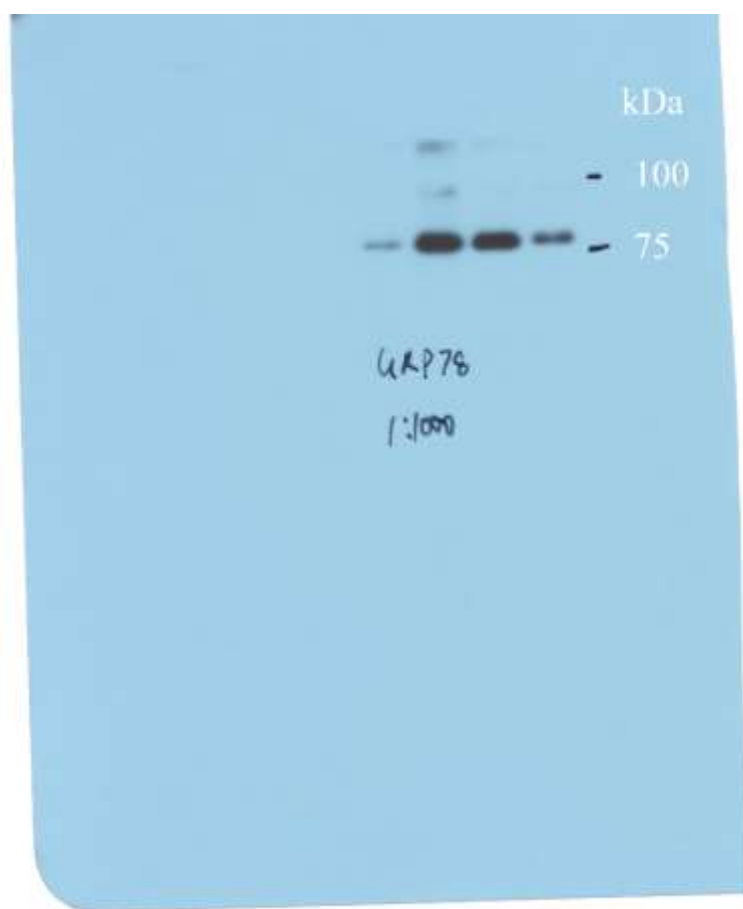

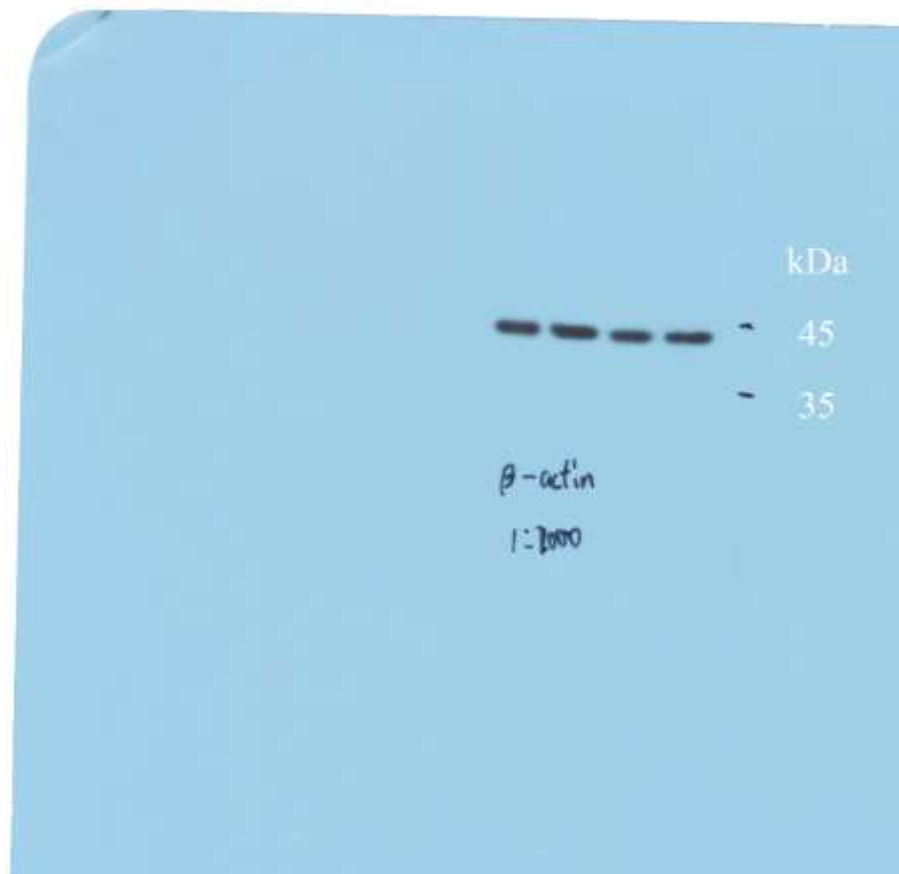

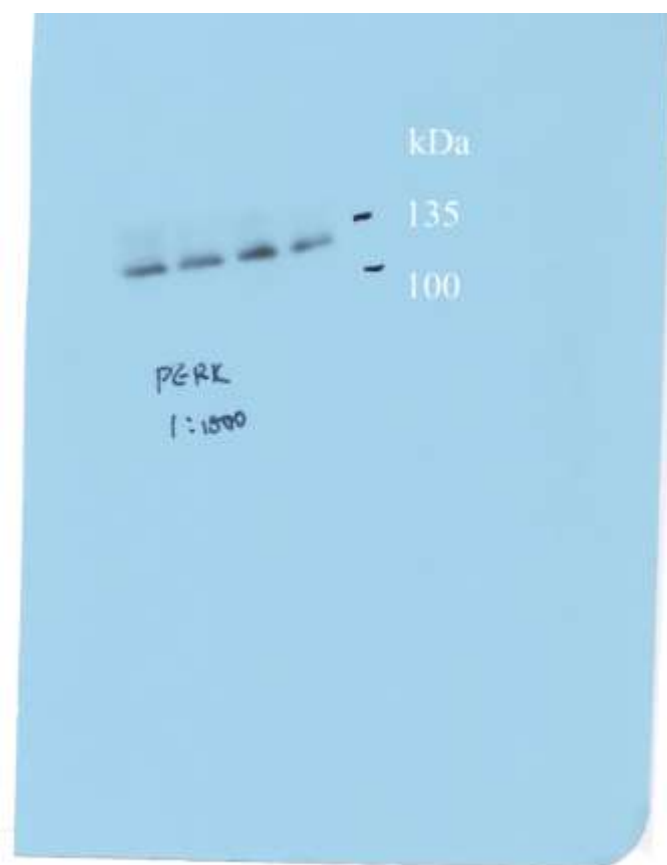

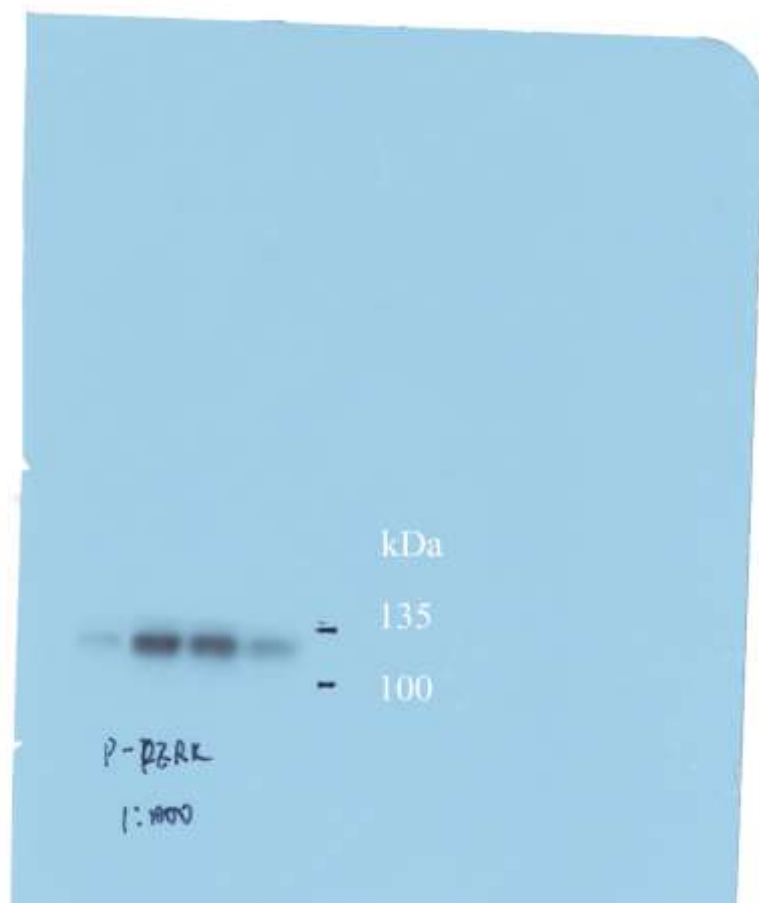

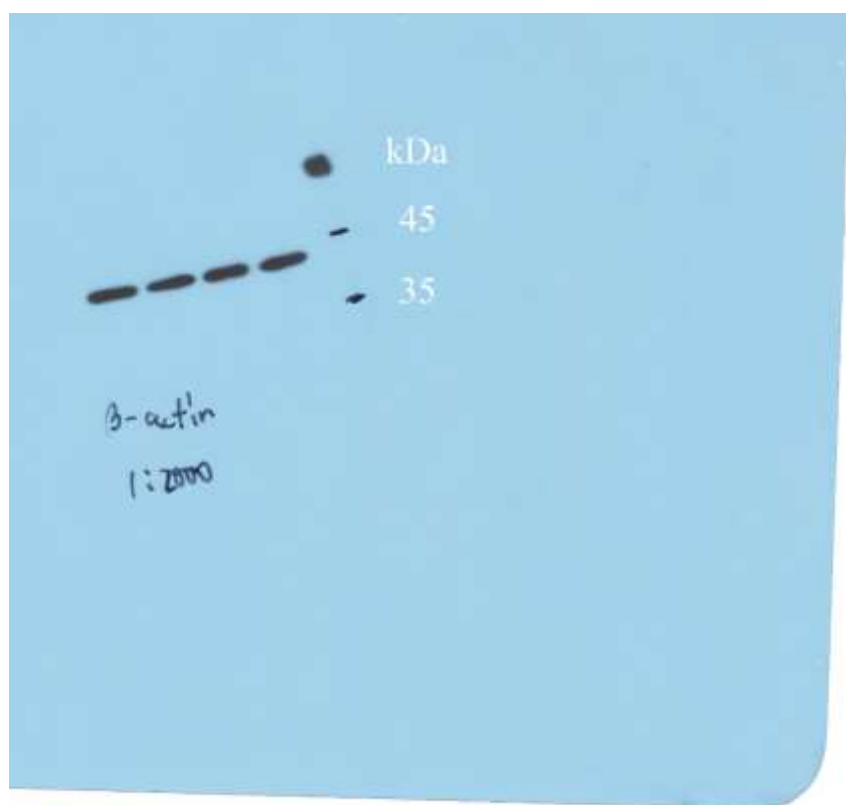

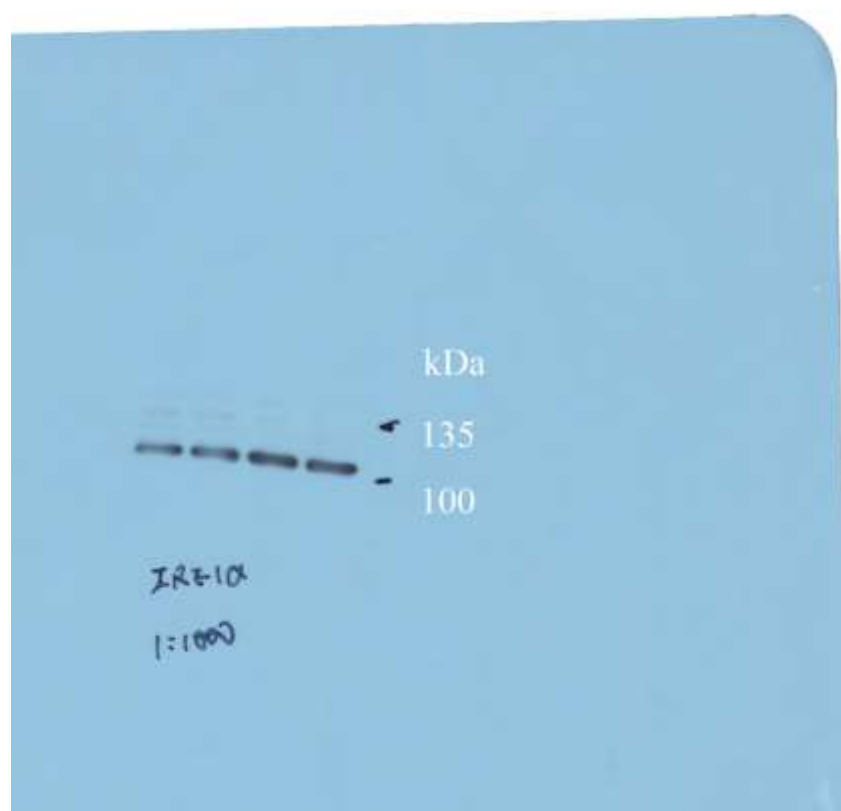

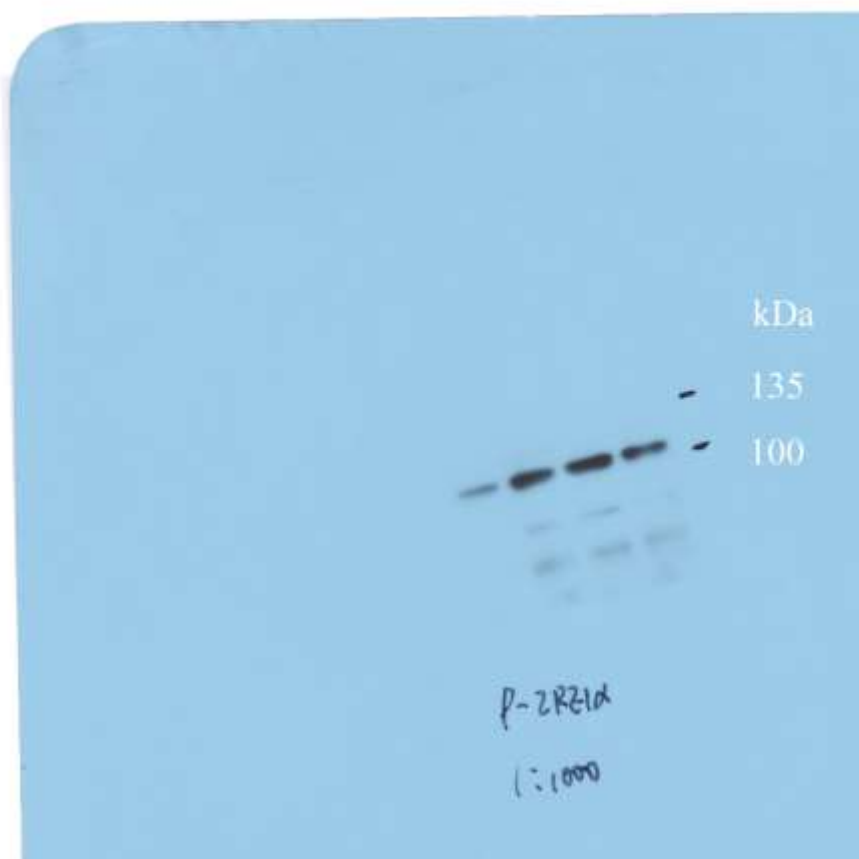

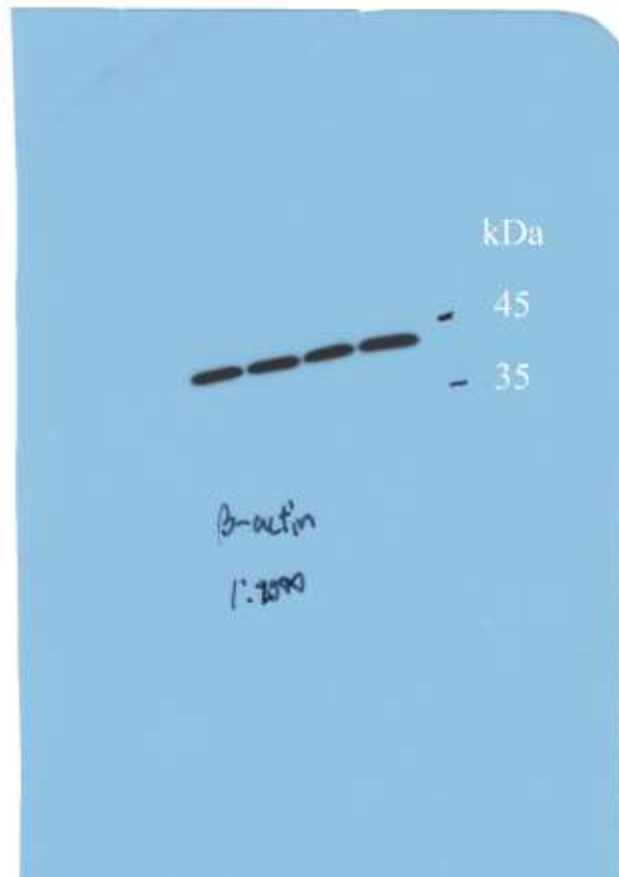

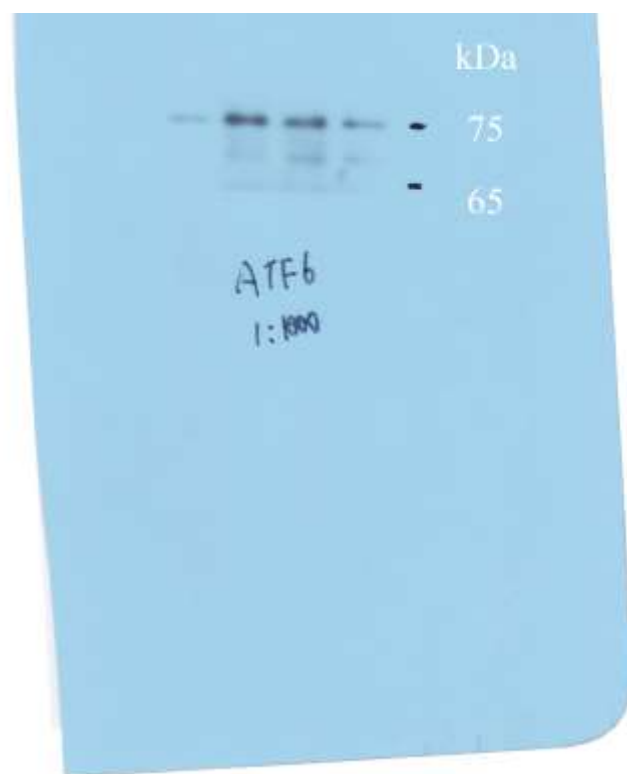

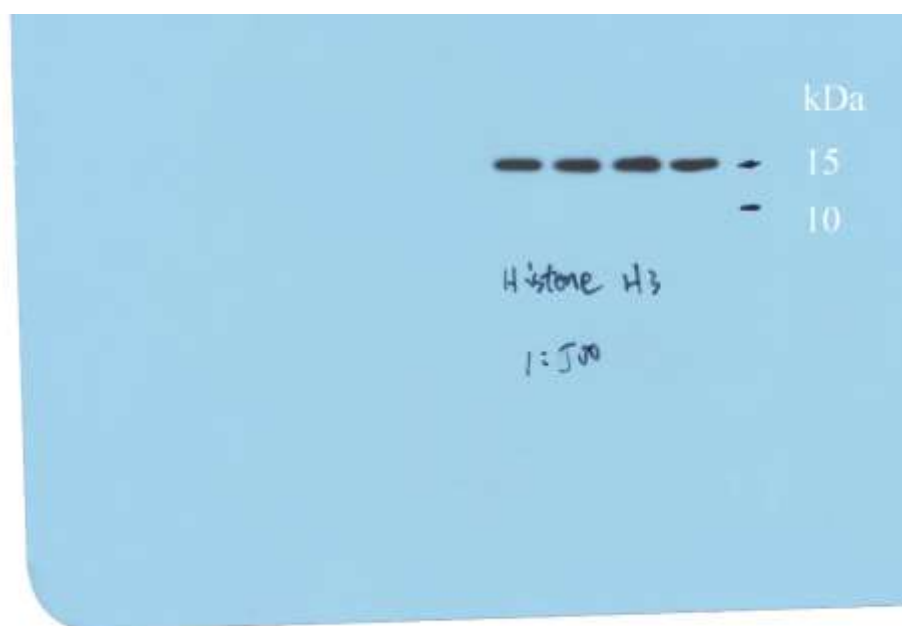

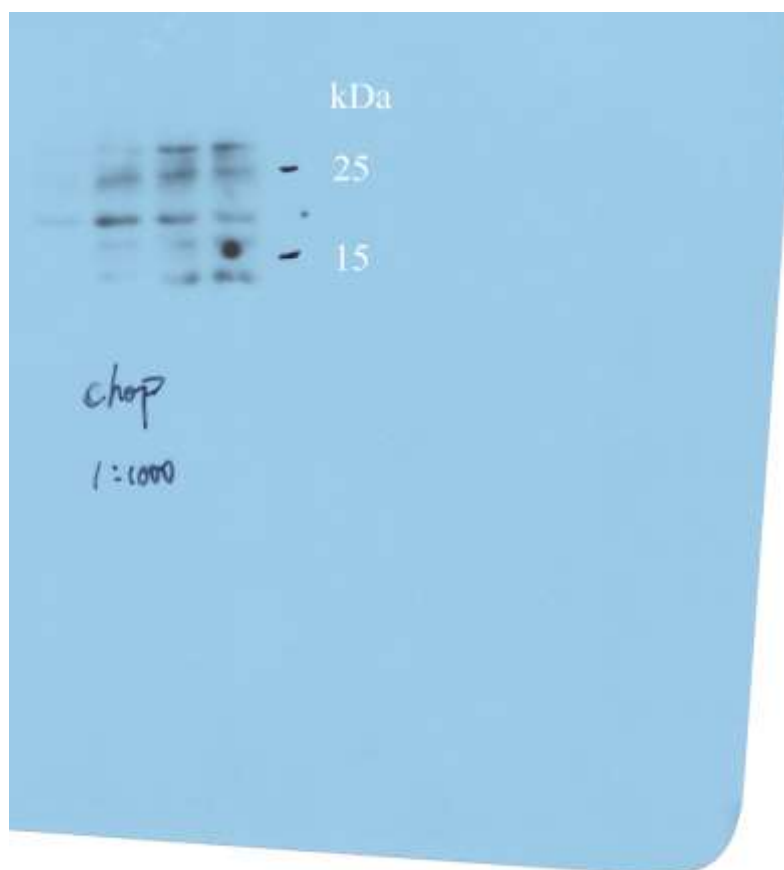

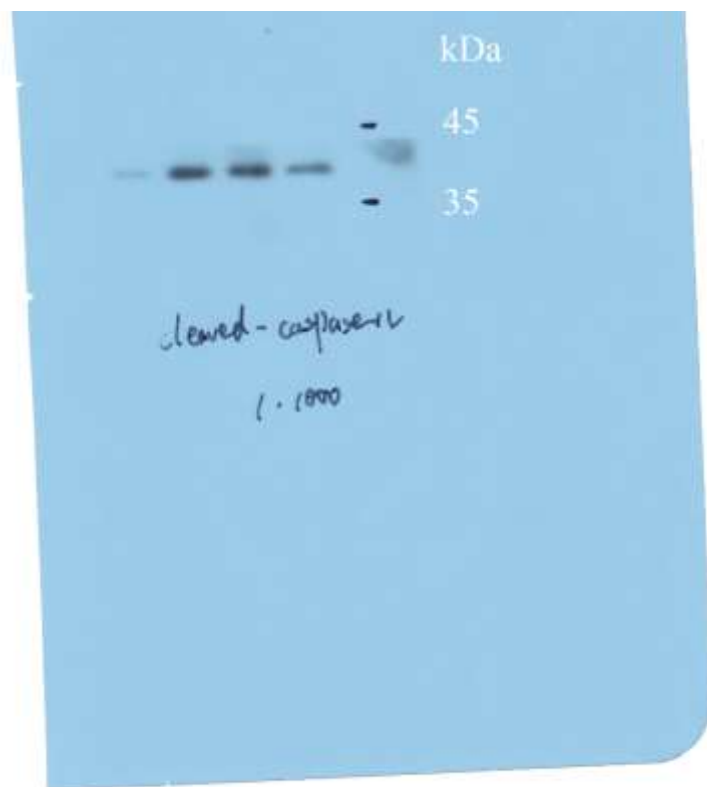

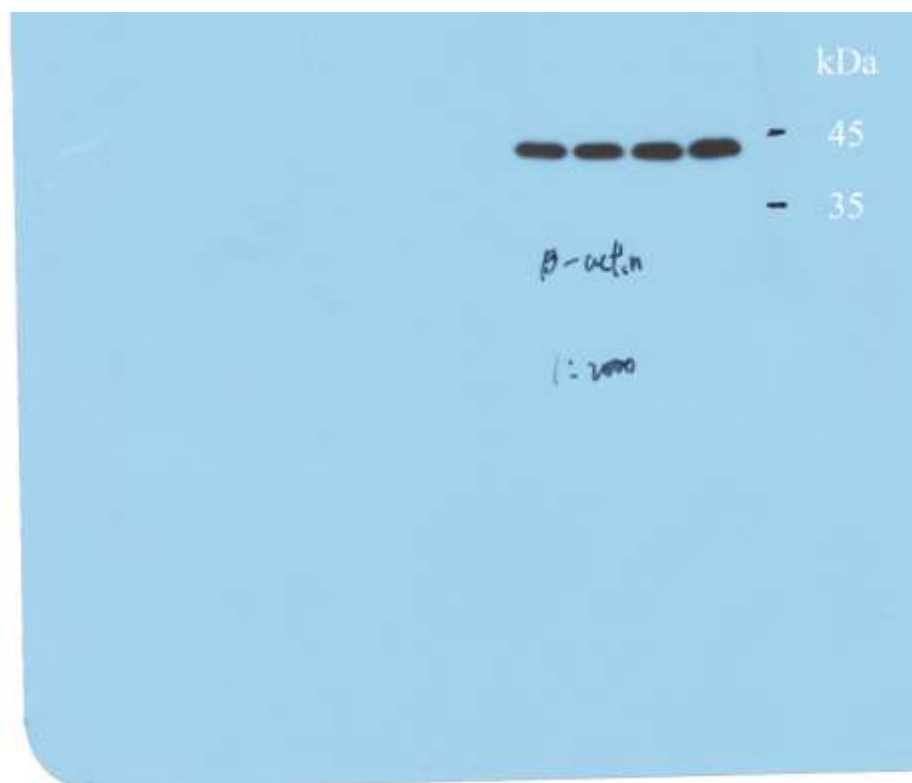

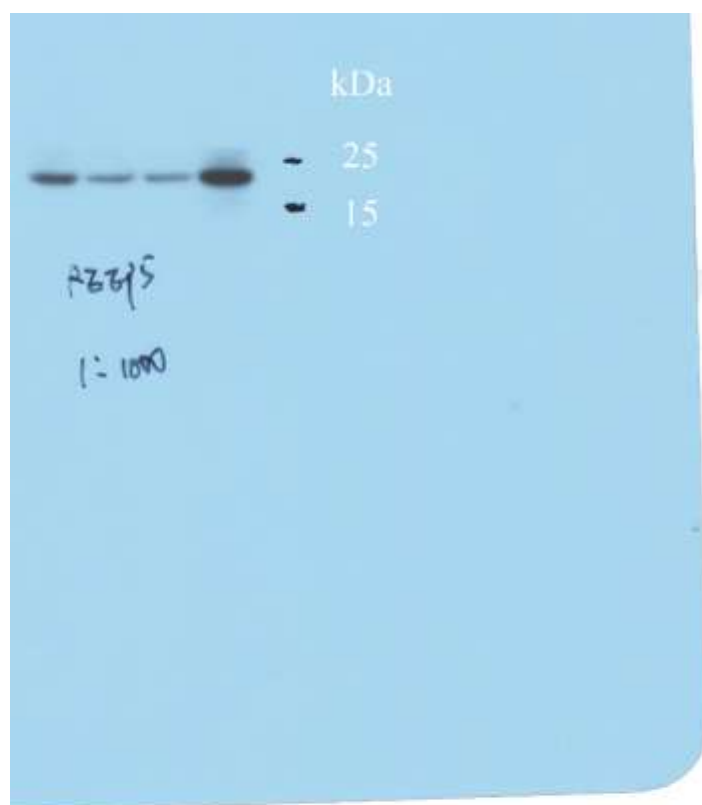

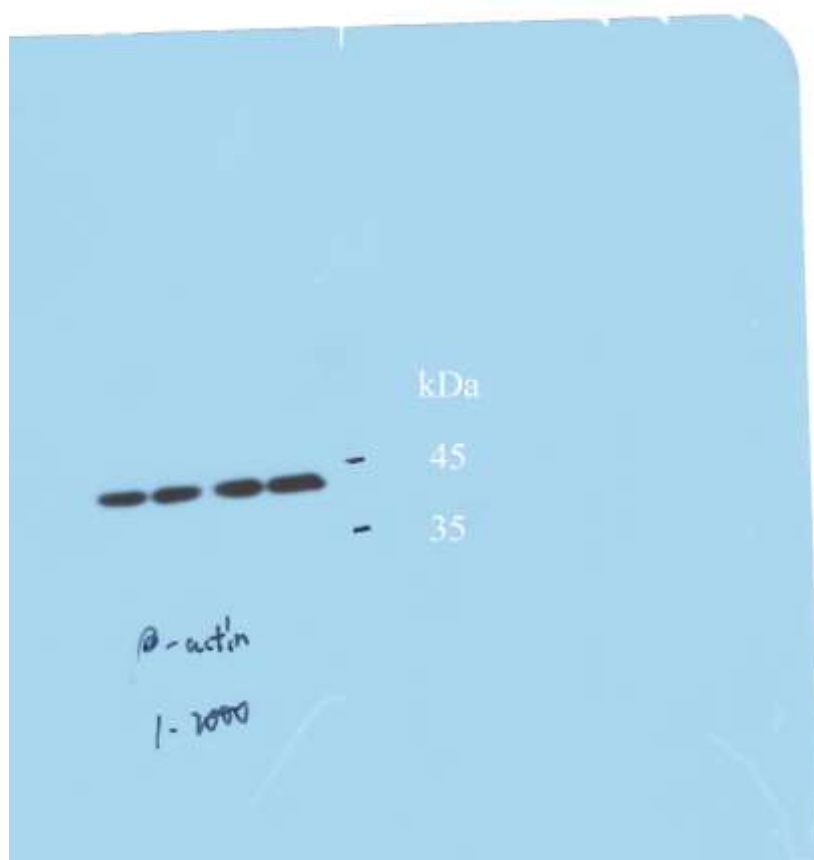

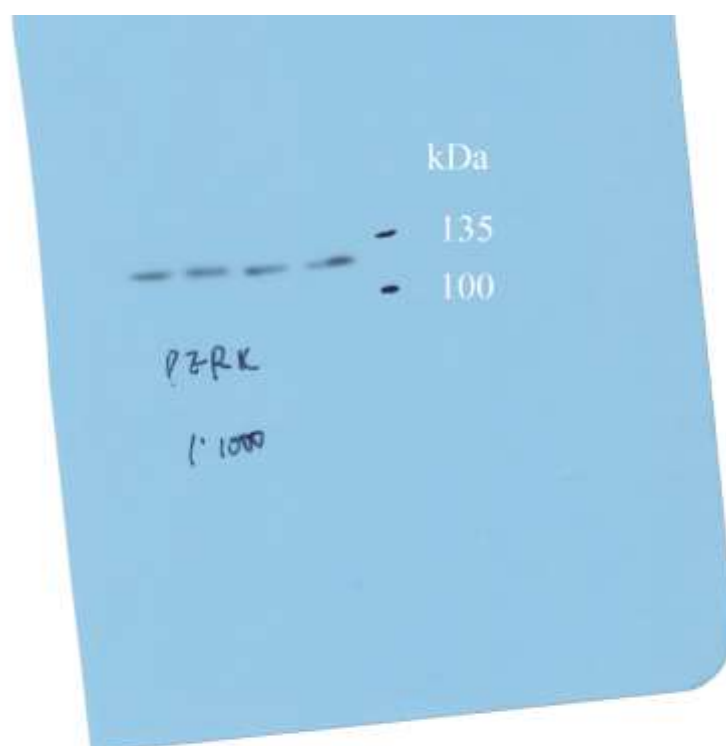

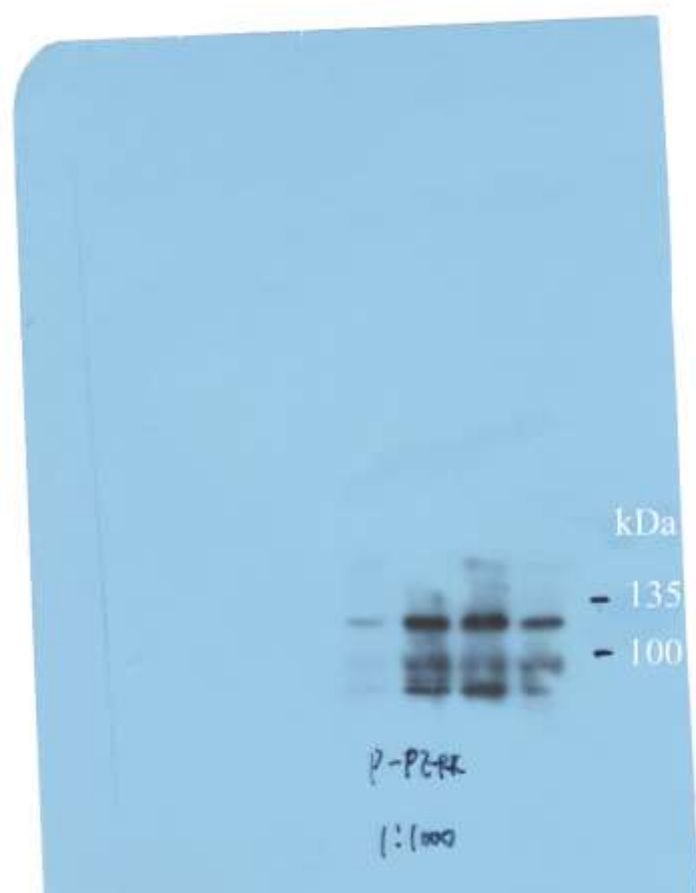

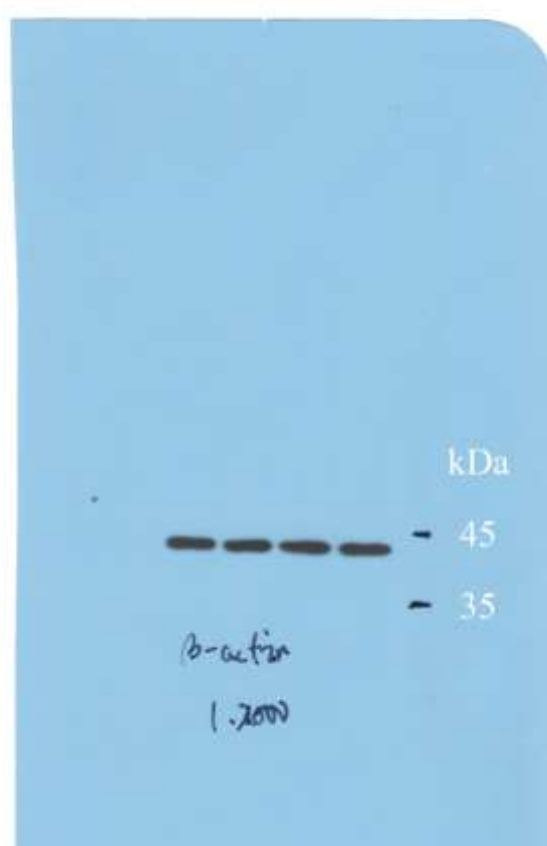

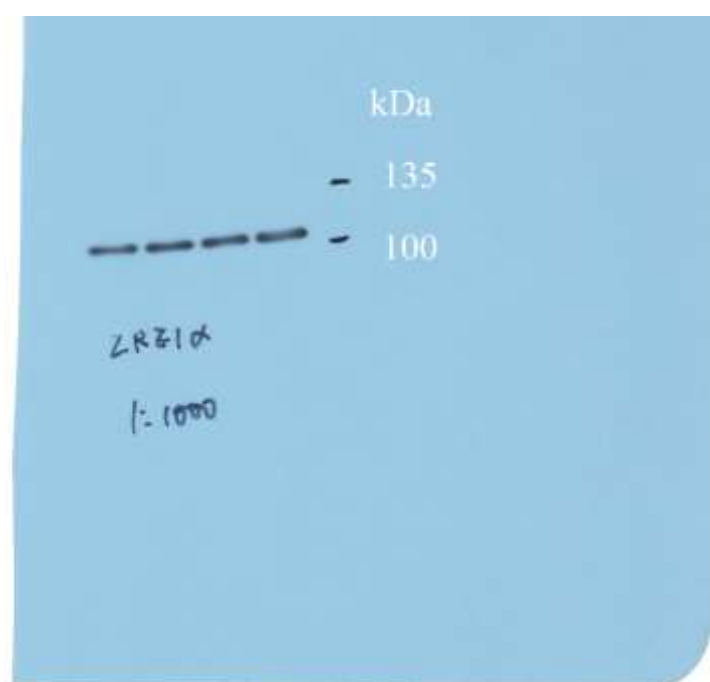

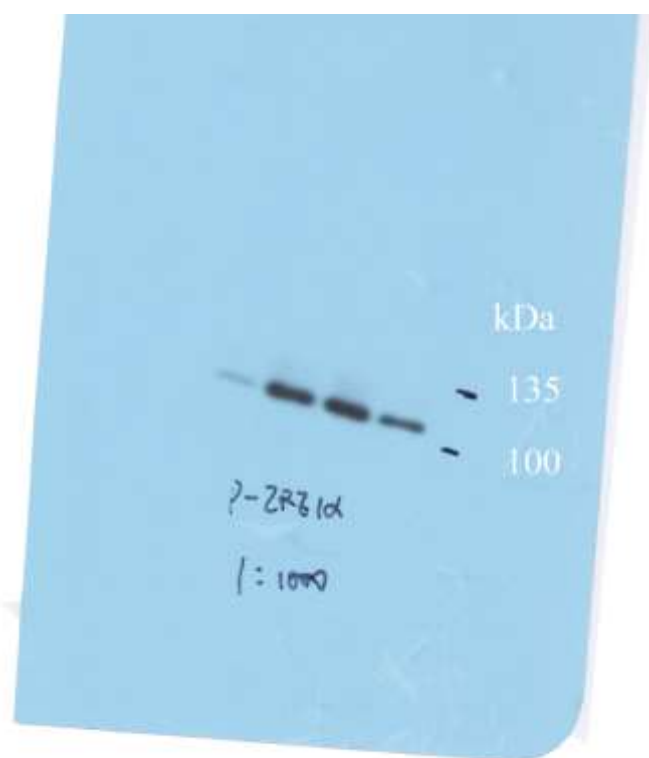

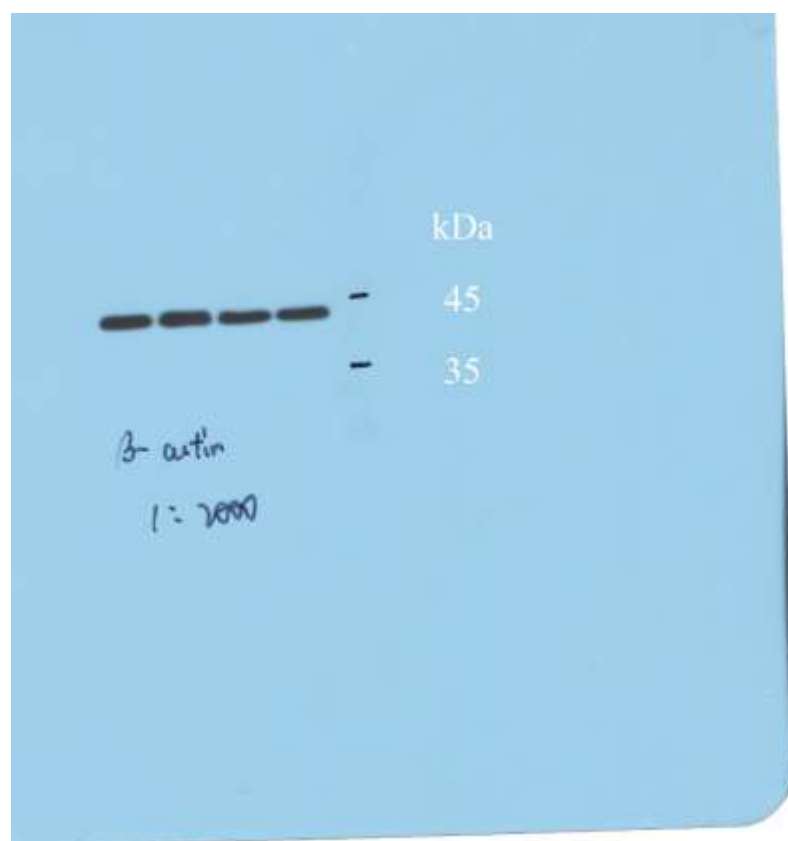

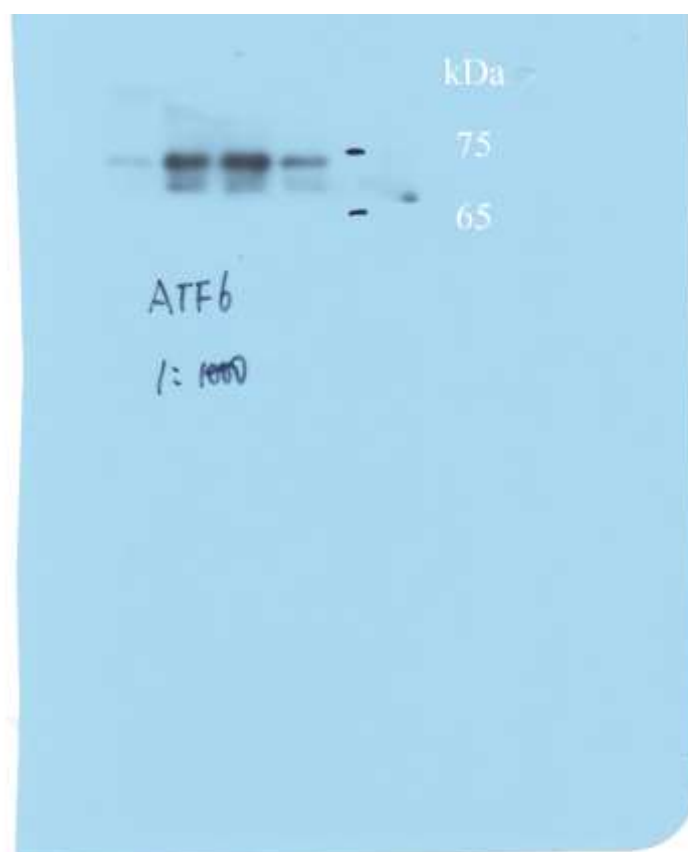

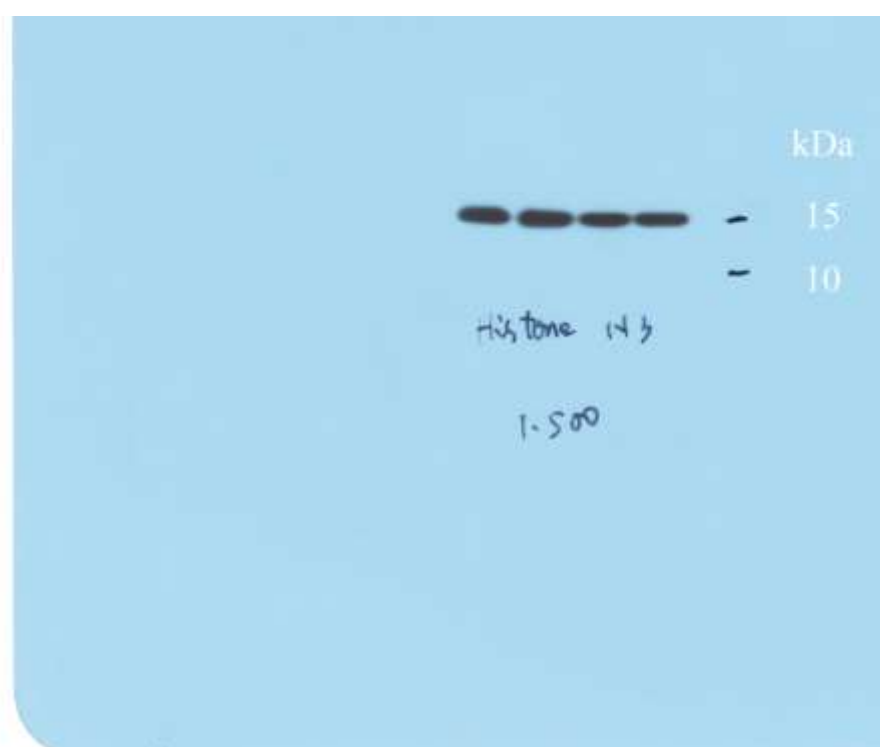

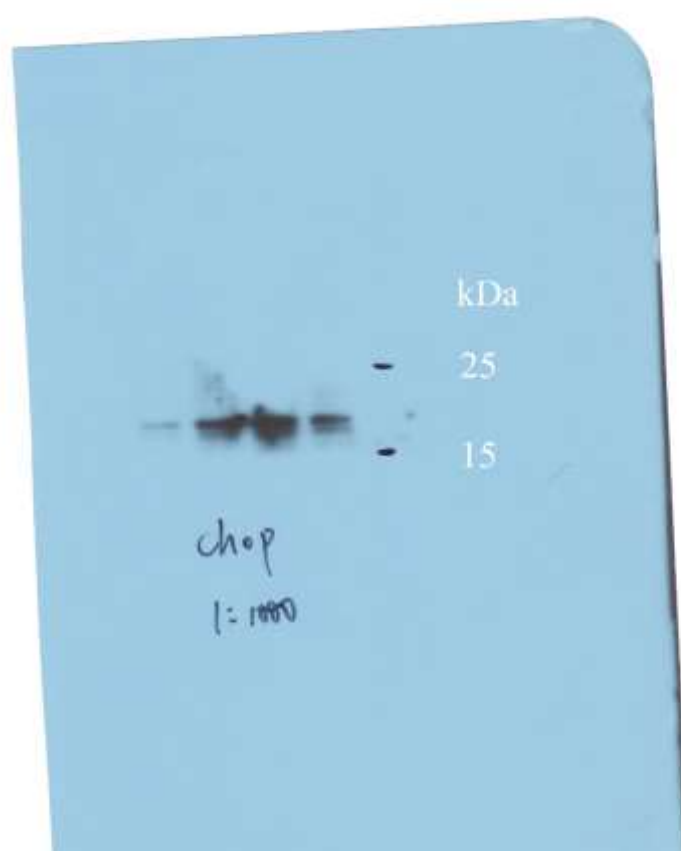

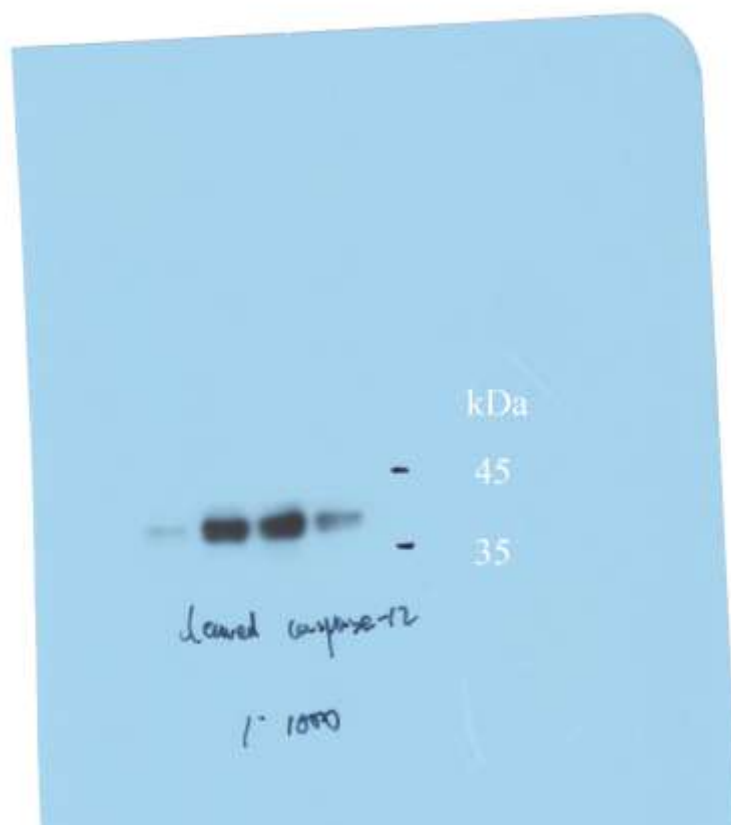

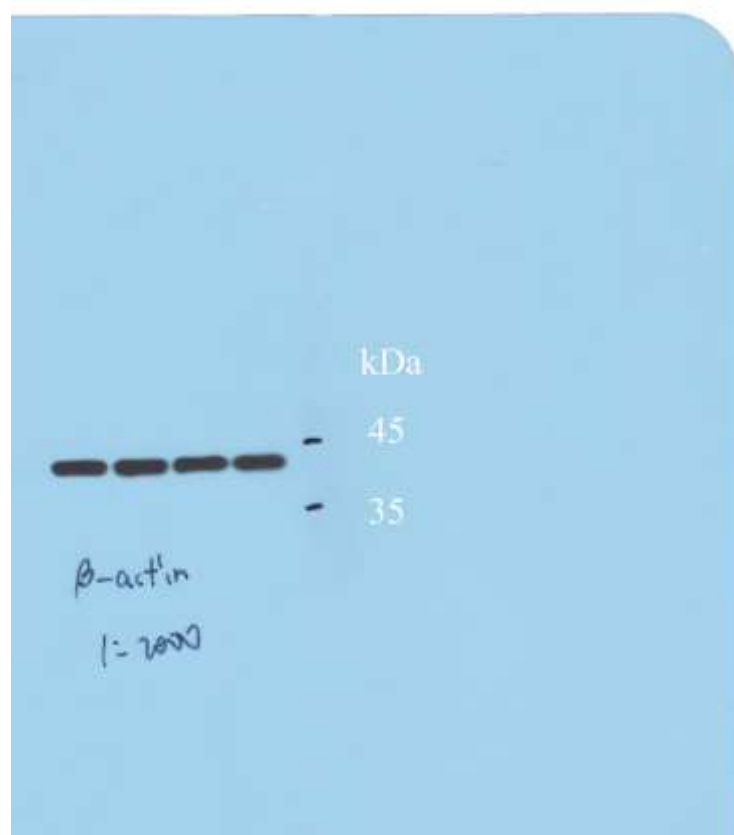

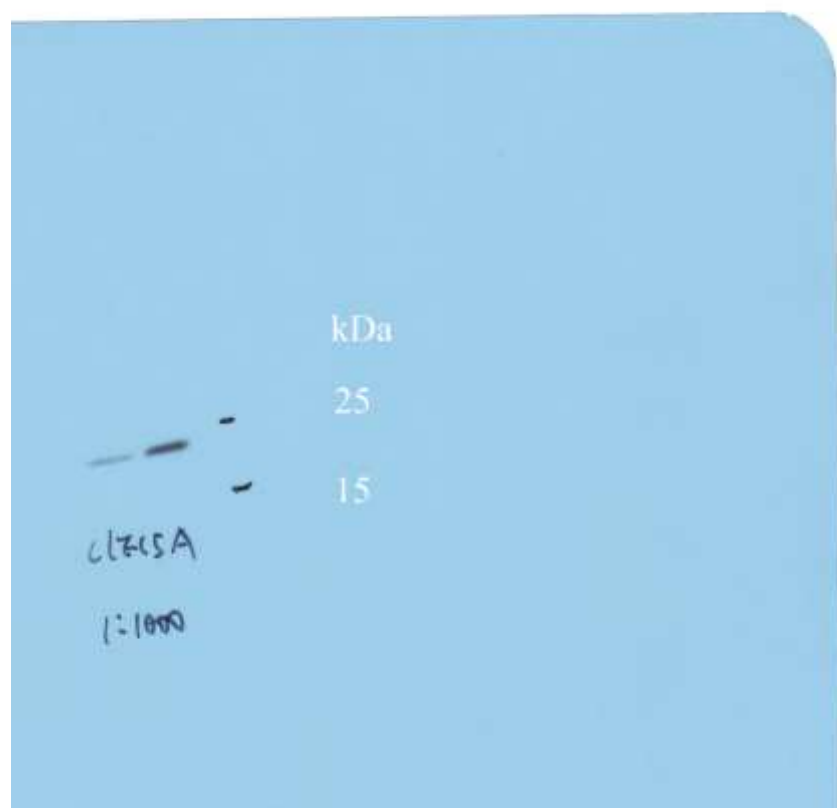

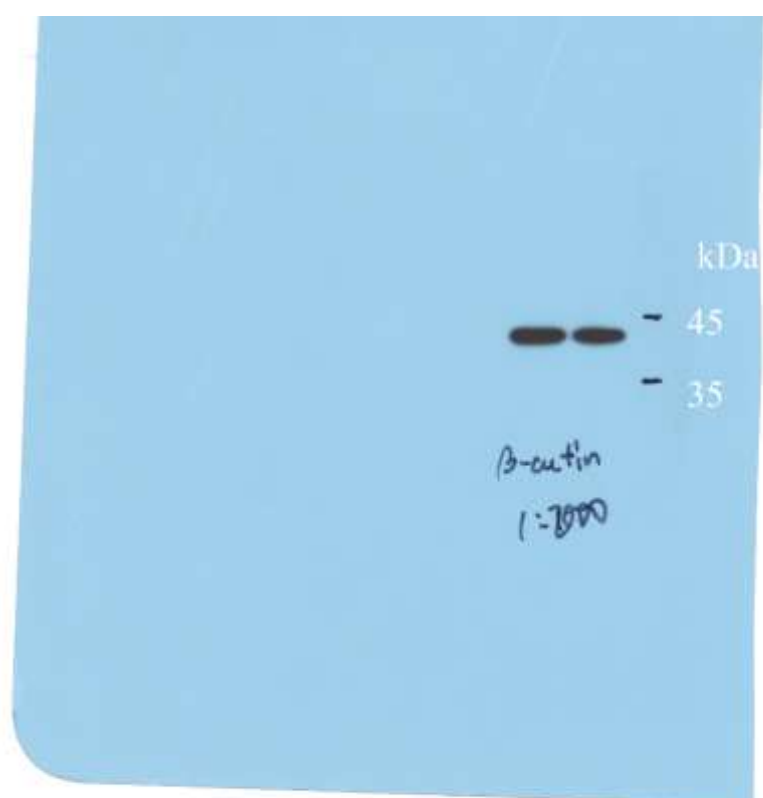

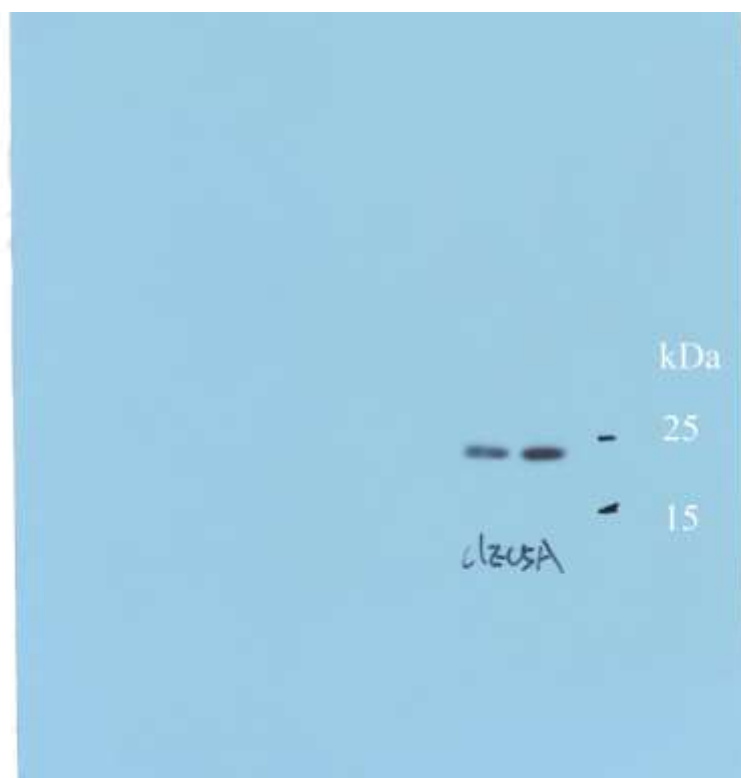

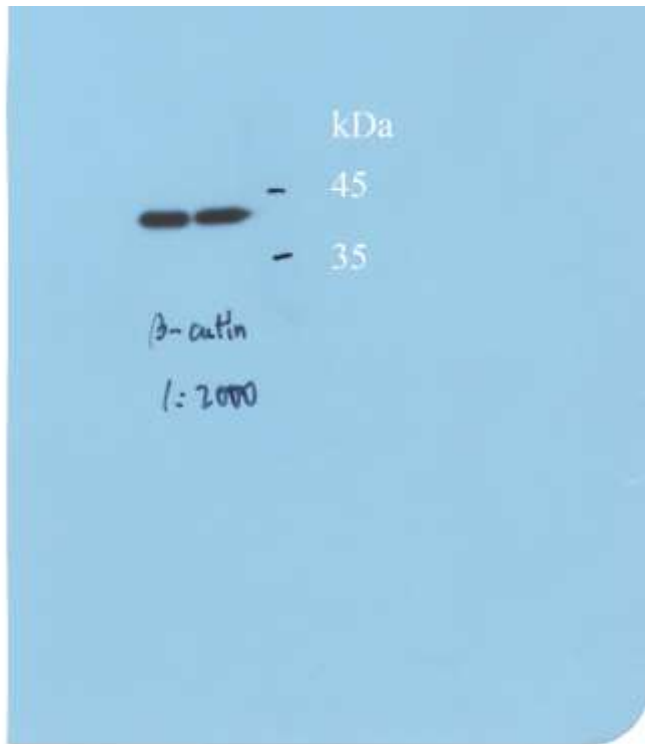

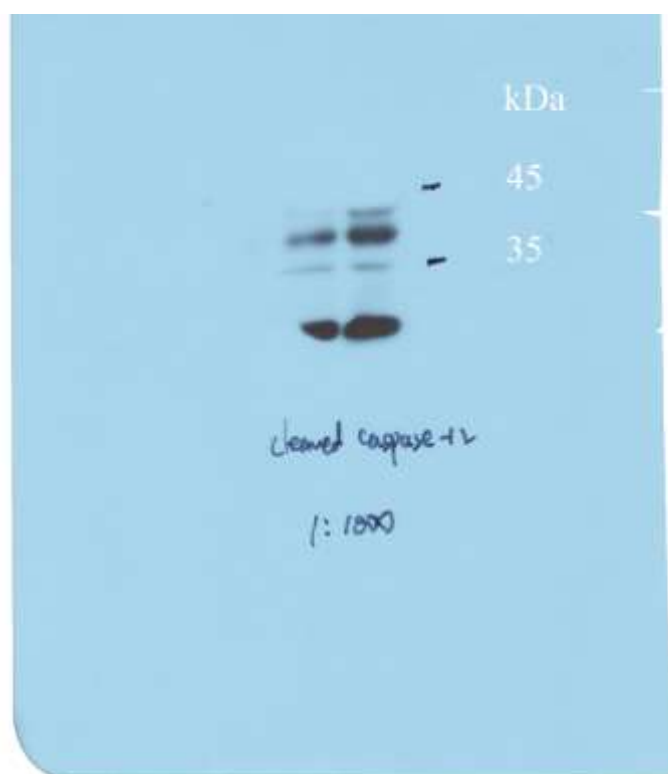

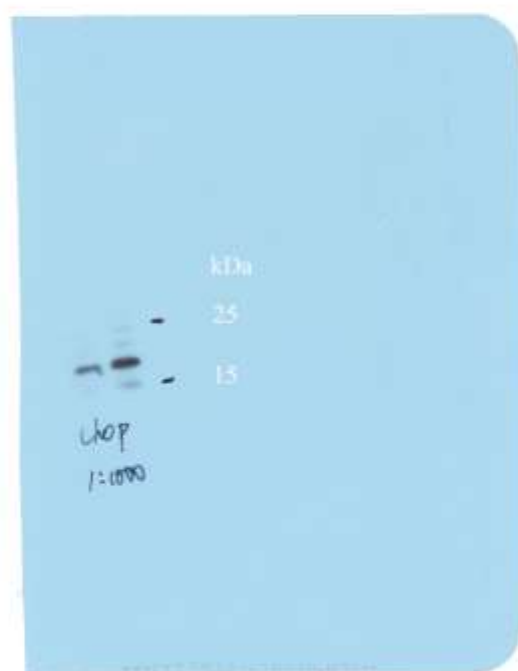

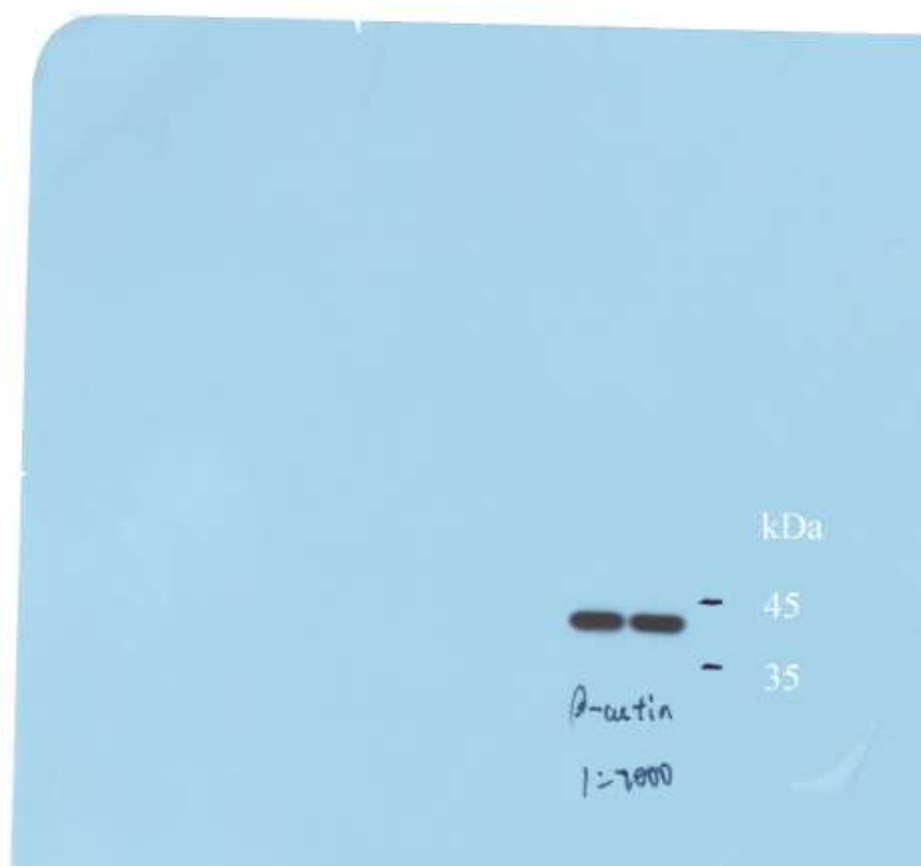

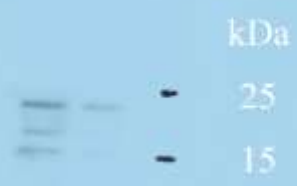

R2GpS

1:1000

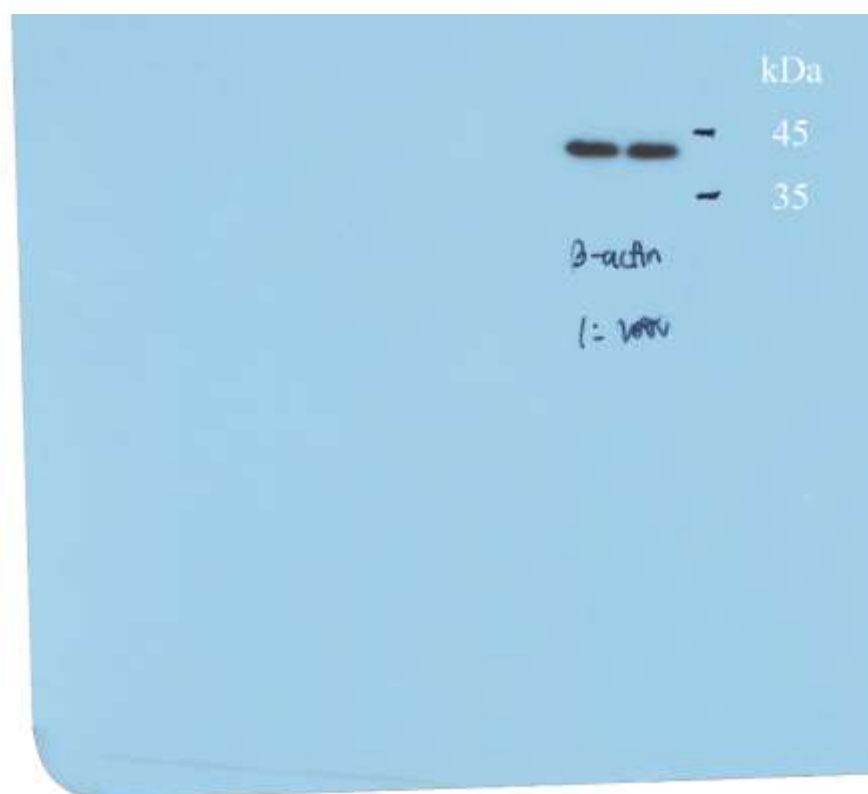

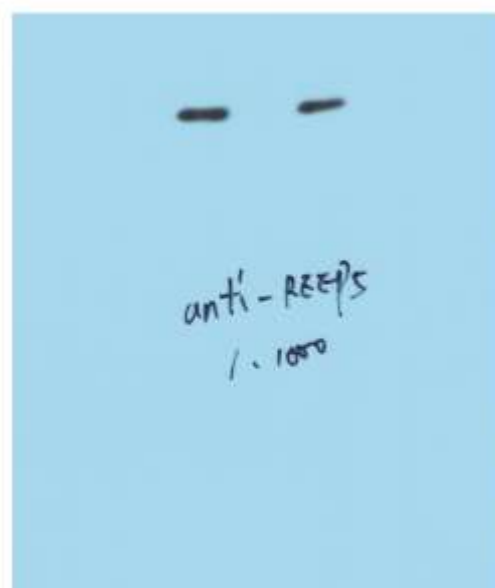

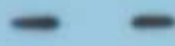

anti-clz5A

1: 1000

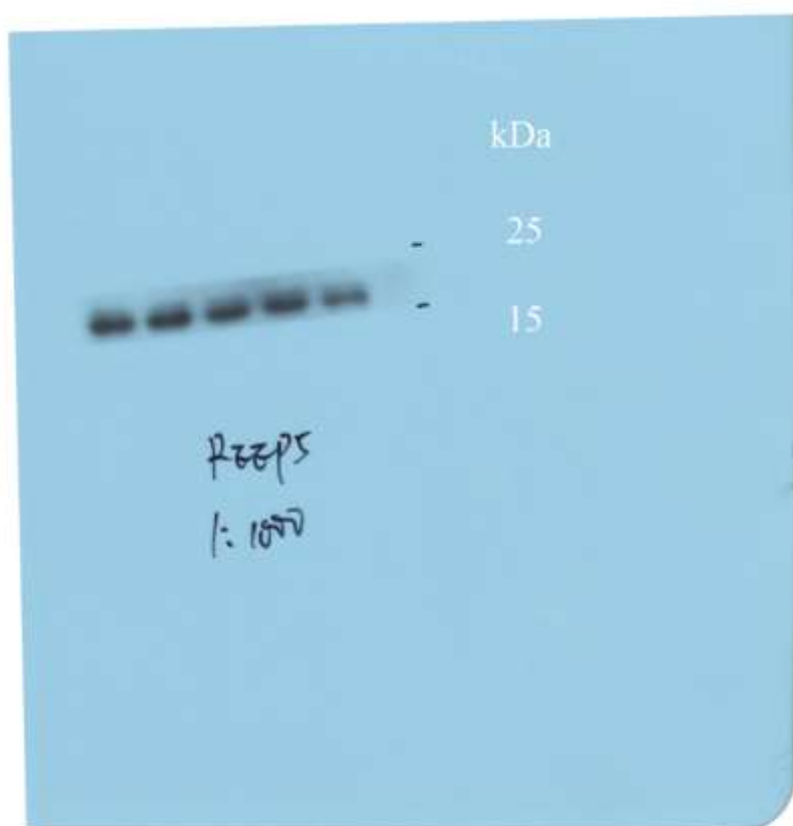

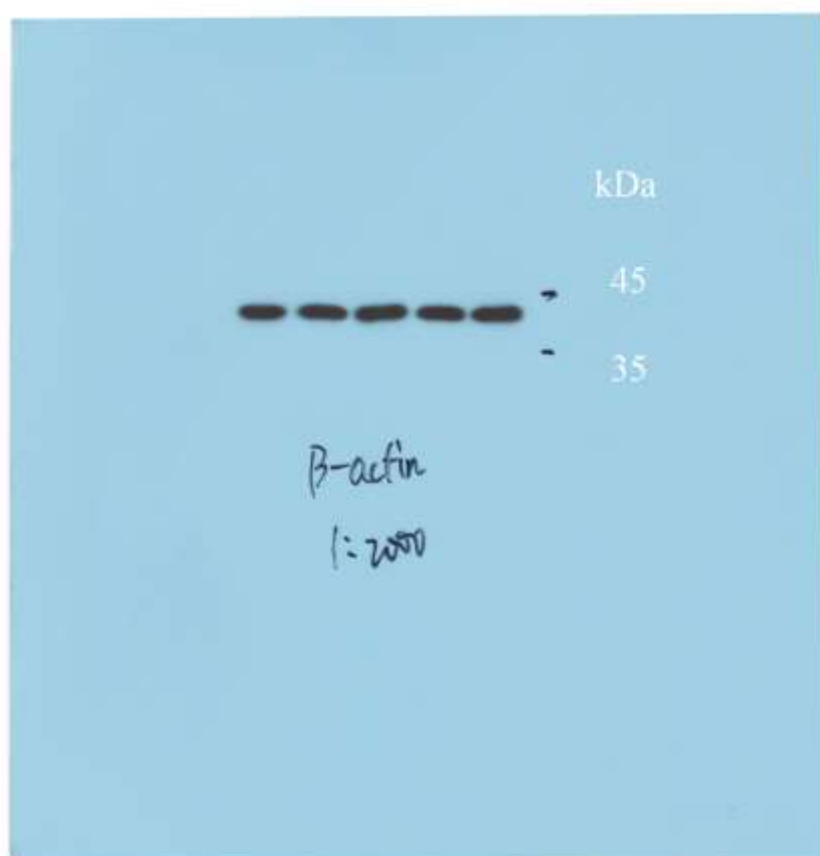

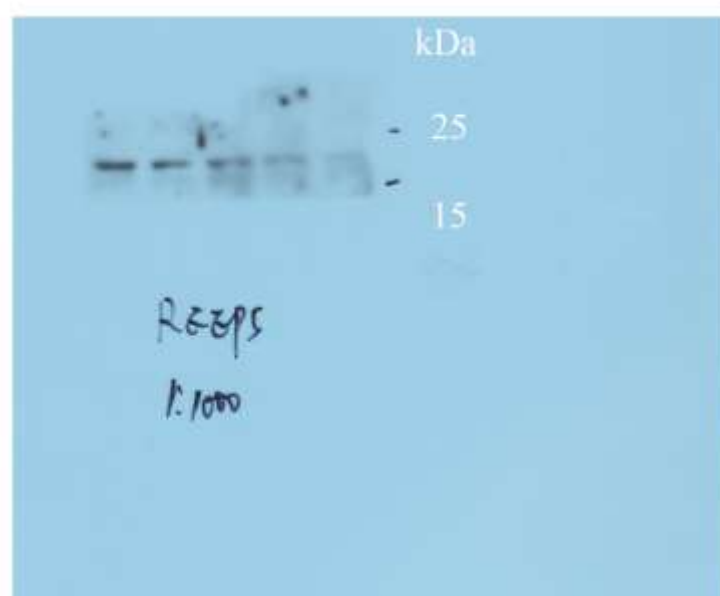

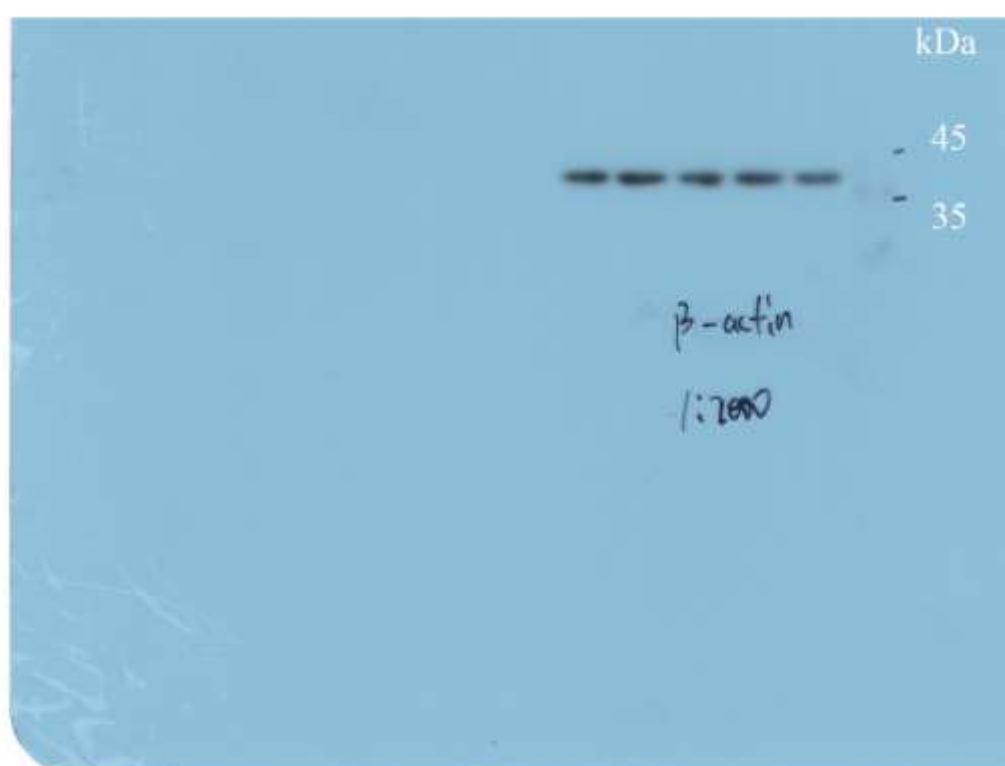

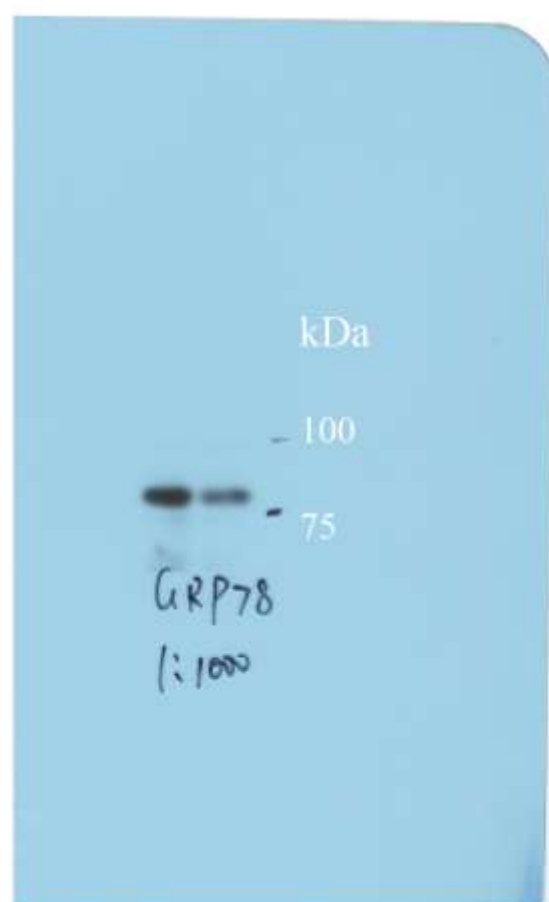

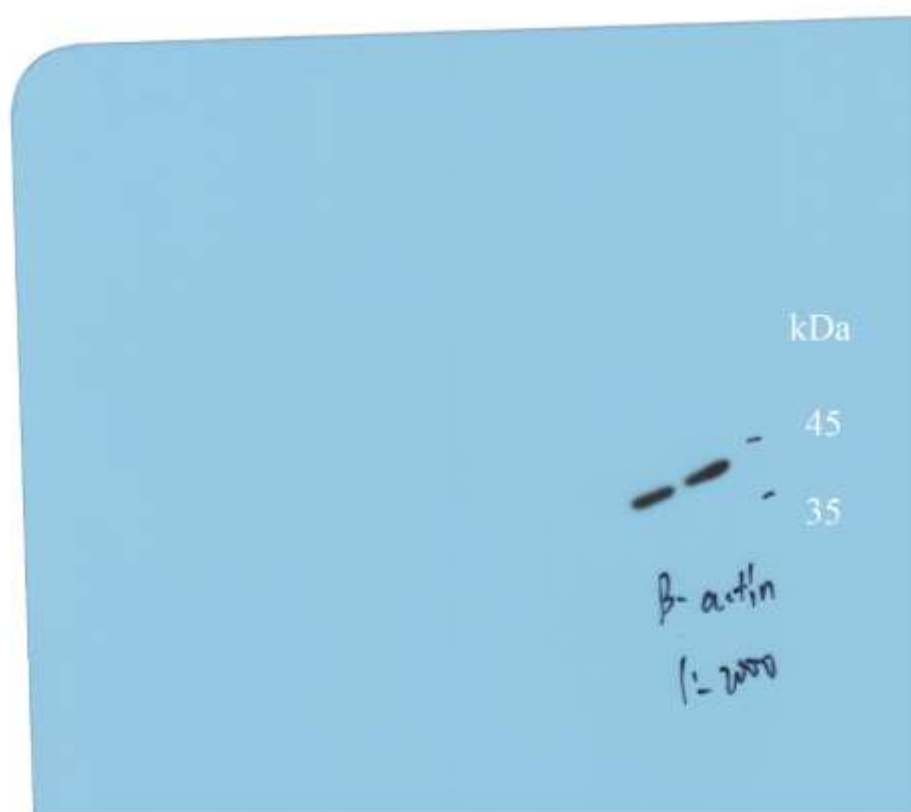

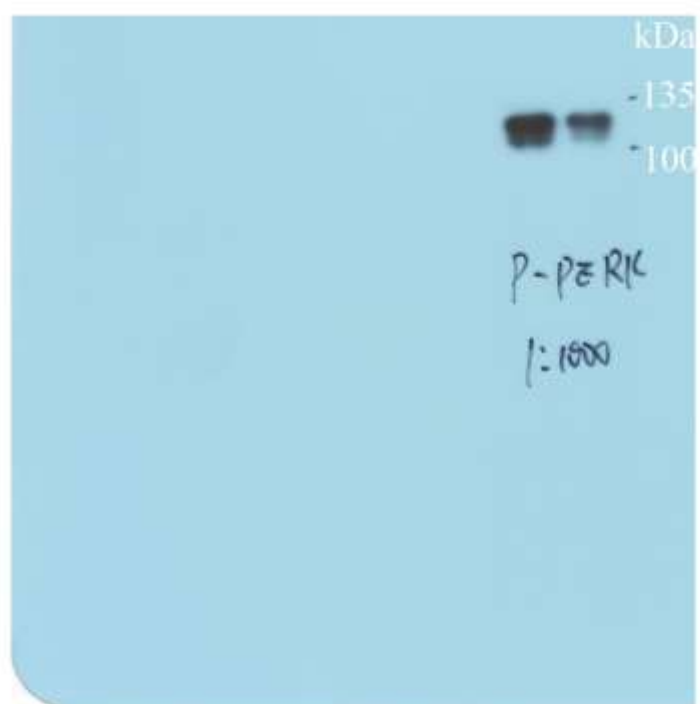

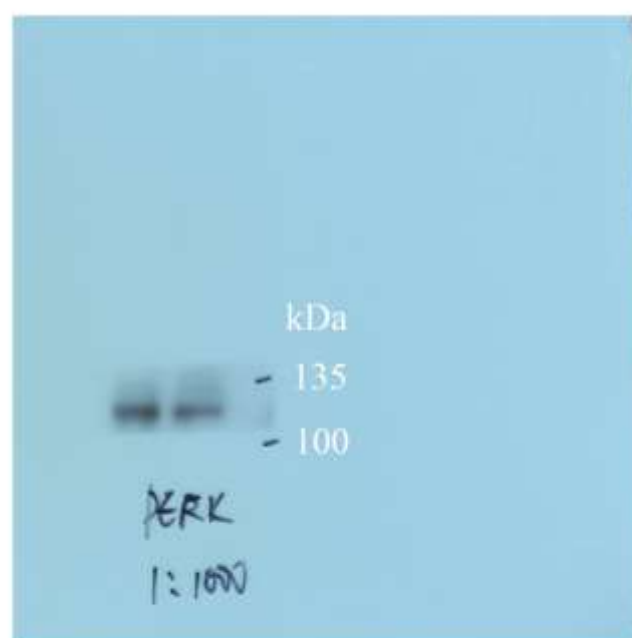

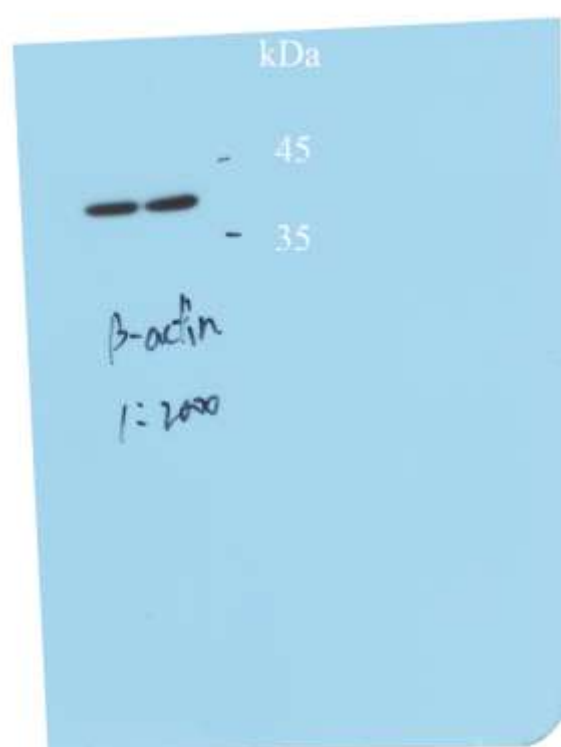

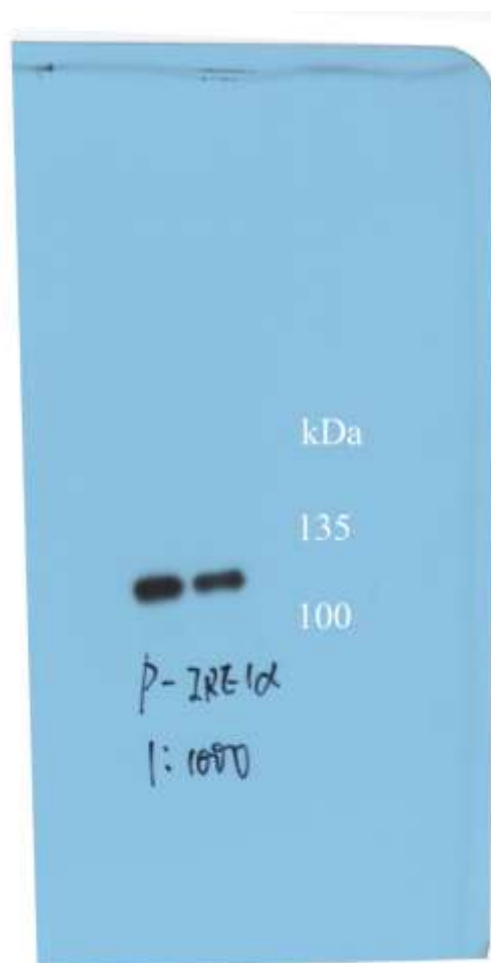

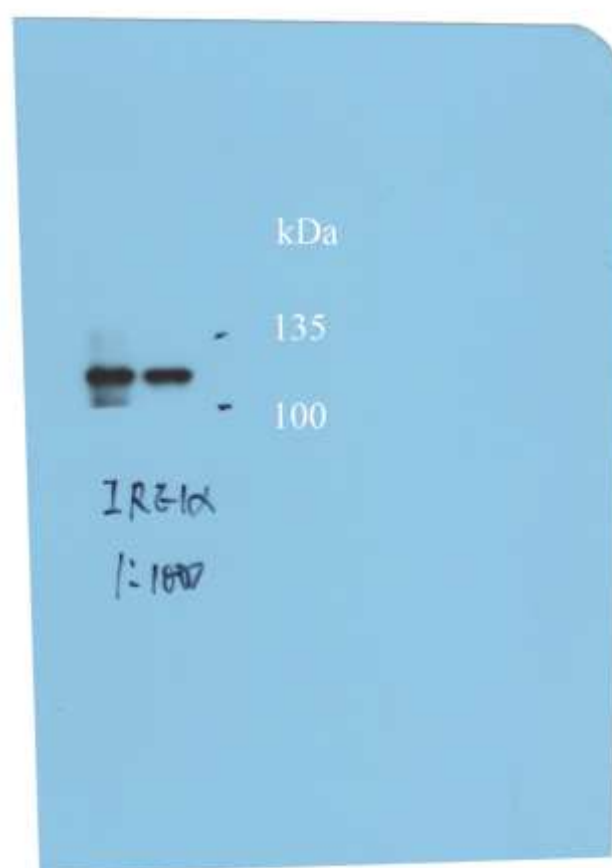

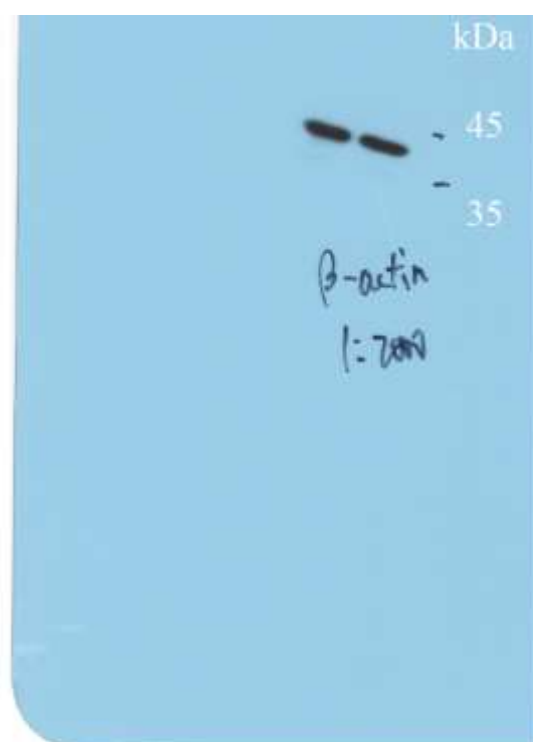

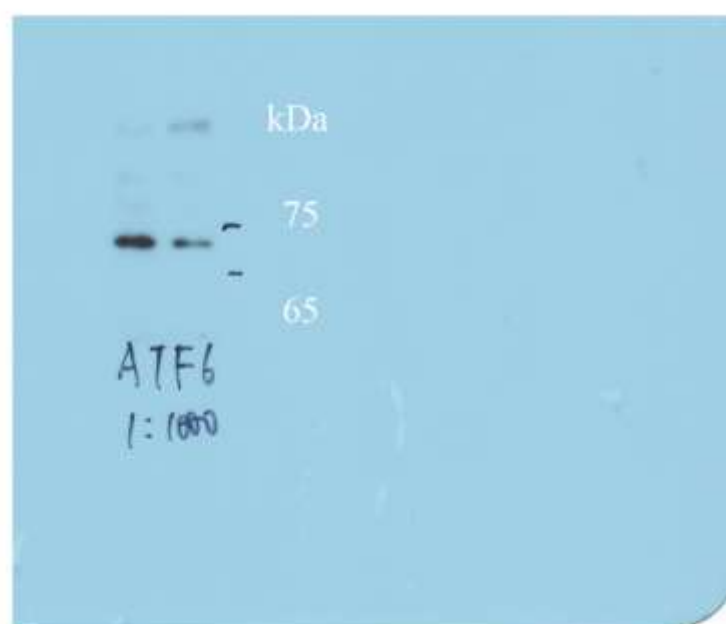

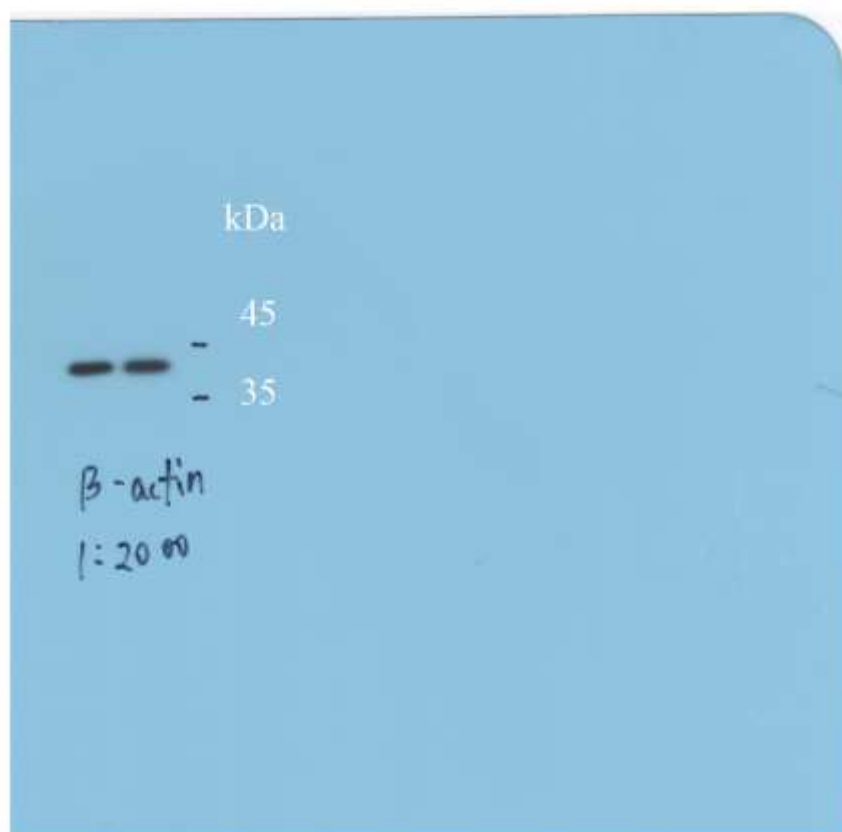

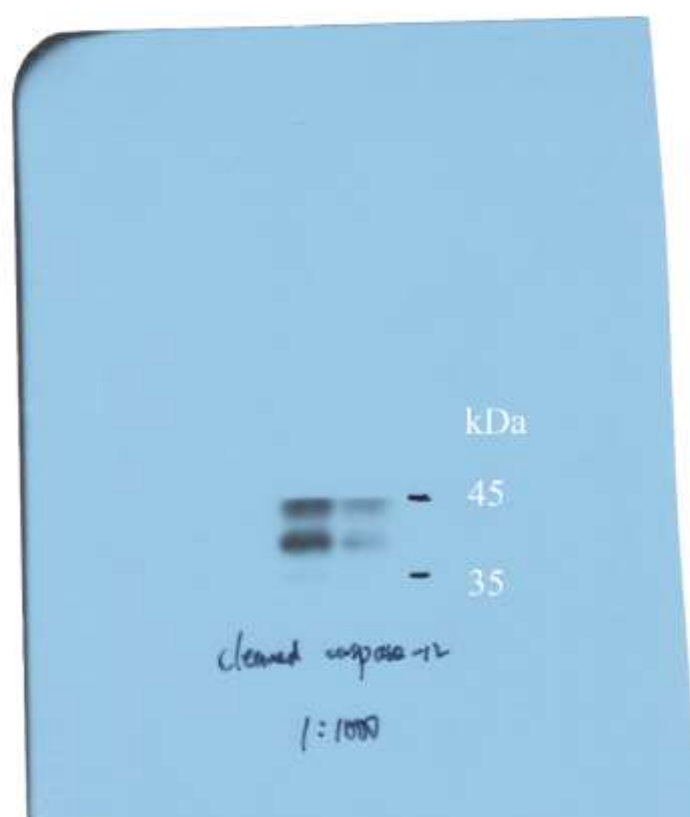

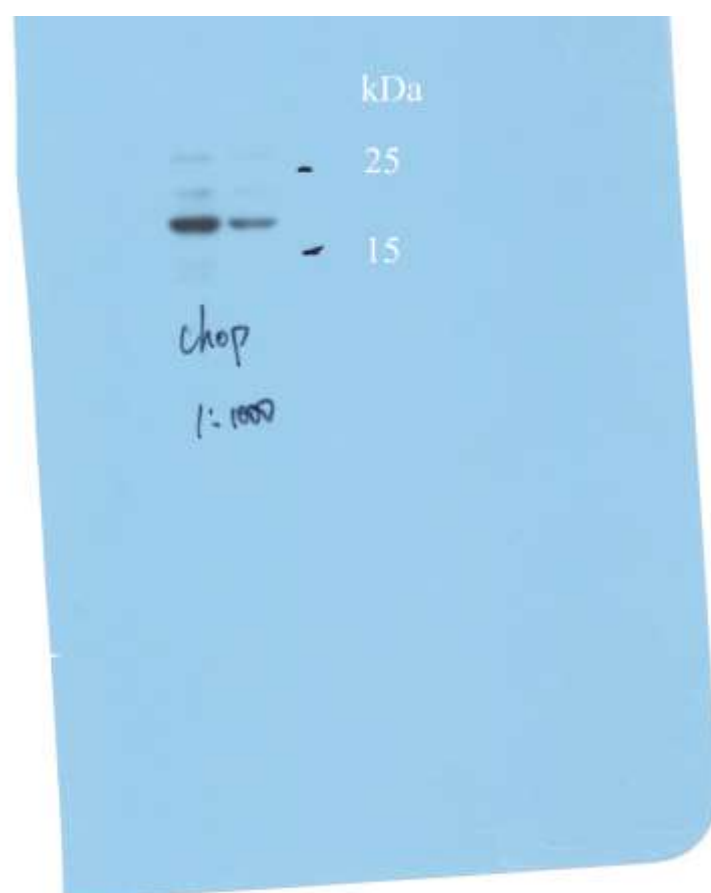

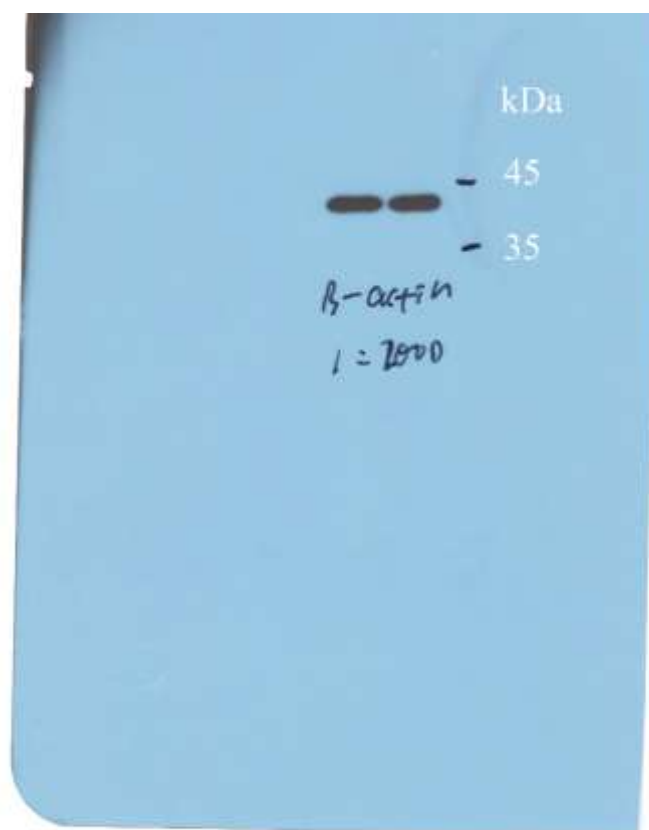

Supplement: Supplementary file 2 — Supplementary Material 2 [file 12872_2024_4018_MOESM2_ESM.pdf]
